# Chondroitin Sulfate Supplementation Is Associated with Body Weight Control in Ovariectomized Rats: A Multi-Omics Study of Gut Microbiota and Metabolite Profiles

**DOI:** 10.3390/biom16081095

**Published:** 2026-07-27

**Authors:** Qingshan Shen, Yanli Ma, Chunhui Zhang, Yujie Guo, Xiaojie Qin

**Affiliations:** 1 Henan Key Laboratory of Zhang Zhongjing Formulae and Herbs for Immunoregulation, Nanyang Institute of Technology, Zhang Zhongjing College of Chinese Medicine, Changjiang Road 80, Nanyang 473004, China; 2 School of Food and Health, Henan College of Classical Chinese Medicine, Hansheng Road 1222, Nanyang 473013, China; 3 Key Laboratory of Agro-Products Processing, Ministry of Agriculture and Rural Affairs, Institute of Food Science and Technology, Chinese Academy of Agricultural Sciences, Beijing 100193, China

**Keywords:** chondroitin sulfate, body weight control, ovariectomized rat, gut microbiota

## Abstract

The prevalence of obesity among elderly women is increasing, particularly in postmenopausal populations. This exploratory study investigated the associations between chondroitin sulfate (CS) supplementation and body weight control in ovariectomized (OVX) rats, a preclinical model for postmenopausal physiology. CS supplementation was associated with significantly reduced body weight gain and preserved adipocyte morphology. CS-treated OVX rats exhibited decreased gut microbiota diversity, with reduced abundance of *Photobacterium* and *Rhodococcus* and increased abundance of *Turicibacter*. Metabolomic analysis revealed elevated levels of carbohydrates, fatty acids, and their conjugates, some of which correlated with body weight parameters. Bioinformatic analysis identified enriched KEGG pathways including tyrosine metabolism, glucosinolate biosynthesis and thiamine metabolism. Collectively, these findings provide preliminary correlational evidence in an OVX rat model supporting the potential of CS as a food supplement for body weight control, although causal relationships and clinical applicability warrant further investigation in postmenopausal populations.

## 1. Introduction

China has been one of the countries with the largest elderly population in the world [1]. A survey showed that the incidence of osteoporosis in the Chinese population aged 50 and above was 19.2%, with a male incidence rate of 6.0% and a female incidence rate of 32.1%, and postmenopausal osteoporosis is able to reduce women’s quality of life [2]. A growing number of postmenopausal women are being diagnosed with osteoporosis, as estrogen levels significantly decrease after menopause, leading to a rapid increase in bone turnover rate and greater bone resorption than bone formation [3]. This is because once premenopausal women enter menopause, the ovary will stop functioning, and the level of estrogen in the body will decrease dramatically and osteoporosis symptoms will result. Research suggested that complete ovary removal in female rats decreased estrogen and resulted in body weight gain or obesity, while administration of exogenous 17β-estradiol for OVX rats could prevent body weight gain [4,5]. This means that ovarian hormone (such as estrogen) level is related with the change of body weight. Based on the World Health Organization (WHO), obesity imposes risks to health, quality of life and life expectancy.

The human intestine harbors an immense ecosystem of roughly 10^13^ to 10^14^ microorganisms. This microbial community is now widely recognized for its complex contributions to host physiology, while its dysregulation has been closely linked to multiple pathological conditions, including obesity, inflammatory bowel disease, and malignancy [6]. In murine models, conventional rearing has been associated with markedly higher adiposity relative to germ-free counterparts; moreover, introducing a normal murine microbiota into germ-free recipients has been shown to elevate total body fat by as much as 60% [7,8]. Body weight can be modulated by alterations of the gut microbiota through administration of non-starch polysaccharide or probiotics and prebiotics [9]. For instance, the predominance of Firmicutes and reduction of Bacteroidetes could influence the increased energy to control body weight [10]. Additionally, estrogen exerts a regulatory influence on the composition of microbiota across multiple body sites, including the gut, vagina, and oral cavity. In menopausal women, the deficiency of this hormone, coupled with its consequent disruption of the intestinal flora, is now considered a primary driver underlying the concurrent increases in both body weight and lipid storage [11]. The estrogen metabolism is firstly subjected to the conjugation in liver by glucuronidation or sulfation, and then it is excreted back into the intestine [12]. In the intestine, the conjugated estrogen will interact with the gut microbiota [13,14,15]. Gut bacteria that exhibit β-glucosidase activity can catalyze the deconjugation of estrogens, thereby generating forms with increased bioactivity, and the overall diversity of the intestinal microbiota has been shown to correlate with the levels of estrogen-derived metabolites [14]. This means that the interplay of gut microbiota and estrogen exists.

The gut microbiota can be shaped by the indigestible diet, including the polysaccharides [9,16]. Chondroitin sulfate (CS) is a linear, sulfated glycosaminoglycan composed of repeating disaccharide units of D-glucuronic acid (GlcA) and N-acetyl-D-galactosamine (GalNAc) linked by alternating β-1,3 and β-1,4 glycosidic bonds. The GalNAc residues are typically sulfated at the C-4 or C-6 positions, conferring a high negative charge density and strong polyanionic nature to the molecule. CS is mainly extracted from the cartilage of terrestrial animals and bony fish as the active ingredient of food supplements [17]. As a macromolecule with a molecular weight ranging from 10 to 70 kDa (the CS used in this study was approximately 16.37 kDa), CS exhibits high hydrophilicity, high viscosity, and limited passive permeability across the intestinal epithelium. Consequently, the bioavailability of CS is very low through oral administration, and the majority is degraded by the microbiota in the gut and simultaneously the intestinal flora can be altered by CS [18,19]. Currently, some research has reported that CS could prevent gaining in body weight and ameliorate obesity by modulating gut microbiota [20,21]. In addition, its oligosaccharides reduced obesity by regulating lipid disorders in a high-fat diet mouse model [22,23]. However, the effect of CS on the body weight of postmenopausal women is not clear.

To investigate the potential role of CS in postmenopausal weight management, we established an ovariectomized (OVX) rat model by bilateral ovariectomy to mimic the physiological state of postmenopausal women. A multi-omics approach integrating 16S rRNA sequencing and untargeted metabolomics was applied to characterize the changes in gut microbiota and intestinal metabolite profiles following 12 weeks of CS administration. Adiposity was evaluated through body fat percentage and histopathological examination of adipose tissue (H&E staining). Our results may provide exploratory insights for understanding the effect of CS on body weight control in the context of estrogen deficiency, and potentially inform future investigations into the anti-obesity bioactivity of CS, although clinical validation is required before translation to postmenopausal women.

## 2. Materials and Methods

### 2.1. Animals and Experimental Design

Eight-week-old female Wistar rats (Beijing Weitong Lihua Laboratory Animal Technology Co., Ltd., Beijing, China) were housed individually in a specific-pathogen-free animal unit. The environmental conditions included a reverse 12:12 h light/dark cycle and a relative humidity of 50–70%, with unrestricted food and water supply. All animal-related procedures were conducted under the ethical license IFST2026004 granted by the local institutional committee (Institute of Food Science and Technology, Chinese Academy of Agricultural Sciences, Beijing, China), following the EU guidelines 2010/63/EU on laboratory animal welfare.

After one week of acclimation in the animal facility, laparotomy with sham surgery was performed on a subset of six rats, while the other eighteen animals underwent bilateral ovarian resection. The subsequent four weeks were dedicated to postsurgical convalescence, during which no pharmacological interventions were given. The resulting OVX rats were then divided randomly into three groups, each comprising six individuals, and subjected to different regimens: saline vehicle (OVX group), 17β-estradiol at 50 μg/kg (OVX-E group), or CS at a daily dose of 150 mg/kg (OVX-CS group). The CS dose of 150 mg/kg/day was selected based on previous in vivo studies [18], and corresponds to a human equivalent dose of approximately 24.3 mg/kg (about 1460 mg/day for a 60 kg adult) based on body surface area normalization, which is within the clinically used range for CS (800–1200 mg/day). The intervention once a day lasted for 12 weeks. The treatments duration of 12 weeks was chosen to allow sufficient time for CS to exert measurable effects on gut microbiota composition and host metabolic parameters, consistent with previous rodent studies evaluating dietary interventions on microbiota and metabolism. Food intake was recorded randomly on a certain day every week.

### 2.2. Sample Collection and Chemicals

Throughout the experimental period, weekly body weight monitoring was performed on each rat. At the conclusion of the 12-week intervention phase, all animals underwent an overnight fasting period (12 h) prior to tissue collection. The small intestinal contents were aseptically retrieved and split into two aliquots: one destined for microbiome profiling and the other for metabolomics. Meanwhile, the weight of the abdominal adipose tissue was assessed, and representative portions were then preserved in 10% formalin for subsequent histological processing. Following routine paraffin embedding and microtome sectioning, tissue slices were subjected to H&E staining to enable microscopic assessment of adipocyte size and structural integrity. For quantitative morphometric analysis, H&E-stained adipose tissue sections were subjected to panoramic scan. From each group, three rats were randomly selected. For each rat, six non-overlapping microscopic fields were randomly captured, and the cross-sectional area of adipocytes was measured using ImageJ software (ImageJ 1.53a, Wayne Rashband National Institute of Health, USA). The average adipocyte area from the six fields was calculated as the representative value for that individual rat. Data were expressed as mean ± SD (*n* = 3 per group) and compared using one-way ANOVA followed by Duncan’s multiple range tests, with *p* < 0.05 considered statistically significant.

For the bone histomorphometric assessment, right femoral bones were harvested from four randomly chosen rats per group, cleansed of all adherent soft tissues, and preserved in 10% formalin. Visceral organ masses (heart, liver, kidneys, and spleen) were recorded to derive the respective organ coefficients, defined as the percentage of organ weight relative to total body weight. Intestinal luminal contents were stored at −80 °C pending assays. CS employed in this study was procured from Xulang Biotechnology Co., Ltd. (Anda, China), with its production involving enzymatic proteolysis and membrane-based fractionation of bovine cartilaginous material. Structural confirmation of the product as CSA was achieved through FTIR analysis, benchmarked against a commercial bovine cartilage-derived CSA standard (C6737, Sigma-Aldrich, St. Louis, MO, USA). Molecular mass characterization, performed via GPC-MALLS, yielded a value of approximately 16.37 kDa; complete physicochemical specifications are provided as Supplementary Information (Figure S1).

### 2.3. Micro-CT Scanning

For three-dimensional evaluation of femoral trabecular structure, specimens were scanned with a Micro-PET/CT apparatus (Inveon Micro-PET/CT, Siemens, Erlangen, Germany) under the conditions reported in reference [24]. The scanning protocol employed an X-ray source set to 80 kV and 500 μA, delivering a pixel size of 14.93 μm per voxel. To define the trabecular compartment, the region for analysis was initiated at a distance of 2.0 mm from the distal growth plate, with a total of 100 consecutive sections selected in the metaphyseal area. Subsequent quantitative analysis of bone microarchitecture was performed using the scanner’s proprietary software (Inveon Research Workplace version 4.0, Siemens, Erlangen, Germany). The primary endpoints assessed were bone mineral density (BMD), trabecular number (Tb·N), bone volume fraction (BV/TV) and trabecular thickness (Tb·Th).

### 2.4. DNA Extraction, Amplification, and Library Construction

Bacterial DNA was extracted with the NucleoSpin 96 Soil kit (Macherey-Nagel, Düren, Germany) in compliance with the manufacturer’s recommended procedure. Both the yield and integrity of the extracted DNA were verified by NanoDrop 2000 spectrometry (Thermo Fisher Scientific, Waltham, MA, USA) and agarose gel profiling, after which the DNA was diluted to 1 ng/μL and kept at −20 °C. The V3–V4 region of the 16S rRNA gene was subsequently amplified from the diluted DNA using primers 338F and 806R. Amplicon quality was first assessed by electrophoresis, then purified with VAHTSTM DNA Clean Beads (N411-03, Vazyme Biotech Co., Ltd, Nanjing, China). The resulting purified products were quantified via the Qubit^®^ dsDNA BR assay kit (Q32854, Thermo Fisher Scientific, Waltham, MA, USA) and combined at equal concentrations for sequencing on an Illumina HiSeq 2500 instrument (HiSeq 2500, Illumina, San Diego, CA, USA) operated by CapitalBio Technology Co., Ltd. (Beijing, China).

### 2.5. Bioinformatics Analysis

Raw sequencing data were processed as follows. Paired-end reads were assembled using FLASH (v1.2.11) with a minimum overlap of 10 bp and a maximum mismatch ratio of 20%. Assembled sequences were then quality-filtered using Trimmomatic (v0.33) with a 50 bp sliding window; sequences were truncated when the average quality score within the window dropped below Q20, and reads shorter than 75% of the original length after trimming were discarded. Chimeric sequences were identified and removed using UCHIME (v8.1). The remaining high-quality clean reads were clustered into operational taxonomic units (OTUs) at 97% sequence similarity using UPARSE (v10.0). OTUs represented by less than 0.005% of total reads were filtered out to reduce the influence of sequencing artifacts. Representative sequences for each OTU were annotated using the RDP Classifier (v2.2) against the SILVA database (Release 132) with a confidence threshold of 0.8. For phylogenetic analysis, multiple sequence alignment was performed using PyNAST (v1.2.2), and phylogenetic trees were constructed using the neighbor-joining method. For differential abundance analysis at the genus level between groups, Metastats was employed, which performs a two-tailed *t*-test to obtain raw *p*-values for each taxon, followed by false discovery rate (FDR) correction using the Benjamini–Hochberg procedure. Taxa with an adjusted *p*-value (*q*-value) < 0.05 were considered statistically significant. Alpha diversity indices (Ace, Chao1, Shannon) were compared between groups using the Student’s *t*-test, without FDR correction as these are single-hypothesis tests per index.

### 2.6. Metabolic Profiling of LC-MS/MS

For metabolomics profiling, sample preparation began with the addition of ice-cold methanol at a sample-to-solvent ratio of 1:3 (*w*/*v*), followed by a 30 min incubation at −20 °C to facilitate metabolite recovery. After centrifugation (15,000× *g*, 15 min, 4 °C), the clear supernatant was recovered for LC-MS/MS injection. Pooled QC samples were generated by combining 10 µL aliquots from all test samples. The analytical platform comprised a Thermo Scientific U3000 UHPLC system (Thermo Fisher Scientific, Waltham, MA, USA) interfaced with a Q Exactive Orbitrap mass spectrometer. Metabolite separation was performed on a Hypersil GOLD aQ column (1.9 µm, 2.1 × 100 mm) maintained under standard conditions. The binary gradient employed mobile phase A (10 mM ammonium acetate) and mobile phase B (acetonitrile with 10 mM ammonium acetate). Total run time was 15 min. The first 1 min of the run consisted of 100% phase A, followed by an 8 min linear gradient from 0 to 100% of phase B. After reaching 100%, a stable run with 100% of phase B was sustained for 3 min, and followed by 3 min of 100% phase A to regenerate the column, pumping at 350 μL/min with a column temperature of 35 °C.

LC-MS/MS data were acquired in both positive and negative ion modes. Data processing was performed using Compound Discoverer software (version 3.2, Thermo Fisher Scientific, Waltham, MA, USA), which included noise filtering, retention time alignment, peak extraction, compound correspondence across samples, gap filling, and background subtraction. Compound identification was based on accurate mass (mass error within 5 ppm) and MS/MS spectral matching against the mzCloud and ChemSpider databases. According to the Metabolomics Standards Initiative (MSI) guidelines, metabolites were assigned to Level 2 (putatively annotated compounds). A total of eight quality control (QC) samples, prepared by pooling equal aliquots of all study samples, and five blank samples (containing only extraction solvent) were included in the analytical batch to monitor system performance and background signals. QC samples were interspersed throughout the LC-MS/MS run and used for (i) instrument stability assessment by overlaying TIC chromatograms; (ii) signal drift correction using QC-based robust Loess signal correction (QC-RLSC); and (iii) feature filtering—features detected in less than 50% of QC samples or with RSD > 30% in QC samples were removed. Blank samples were used to monitor background signals from solvents, column bleed, and instrument carryover; features with intensities in blank samples comparable to or higher than those in biological samples were excluded. After filtering, data were normalized using Constant sum normalization for between-sample normalization, followed by Log2 transformation and Auto scaling for within-sample metabolite standardization. Since all samples were analyzed in a single analytical batch, inter-batch correction was not required. Differentially abundant metabolites between groups were identified based on a combination of (i) variable importance in projection (VIP) > 1.0 derived from the OPLS-DA model, and (ii) a two-tailed Student’s *t*-test. The resulting *p*-values were adjusted for multiple comparisons using the Benjamini–Hochberg FDR procedure. Metabolites with a *q*-value < 0.05 were considered significantly changed. KEGG pathway enrichment analysis was performed using Fisher’s exact test, with *p* < 0.05 considered significant.

### 2.7. Statistical Analysis

All results are expressed as mean ± SD. A *p*-value threshold of 0.05 was adopted to denote statistical significance, with values below 0.01 regarded as highly significant. For intergroup comparisons, one-way ANOVA coupled with Duncan’s post hoc test was applied when more than two groups were involved; when only two groups were compared, Student’s *t*-test was employed. Data preprocessing involved Constant sum scaling followed by Auto scaling transformation. To control the family-wise error rate in multiple hypothesis testing, raw *p*-values from non-parametric analyses were subjected to FDR adjustment. The statistical analyses were implemented in *R* (version 4.6.1), whereas multivariate modeling, specifically PCA and OPLS-DA, was carried out using the SIMCA 14.1 software package.

## 3. Results and Discussion

### 3.1. OVX Rat Model to Simulate the Postmenopausal Woman

**Figure 1:**
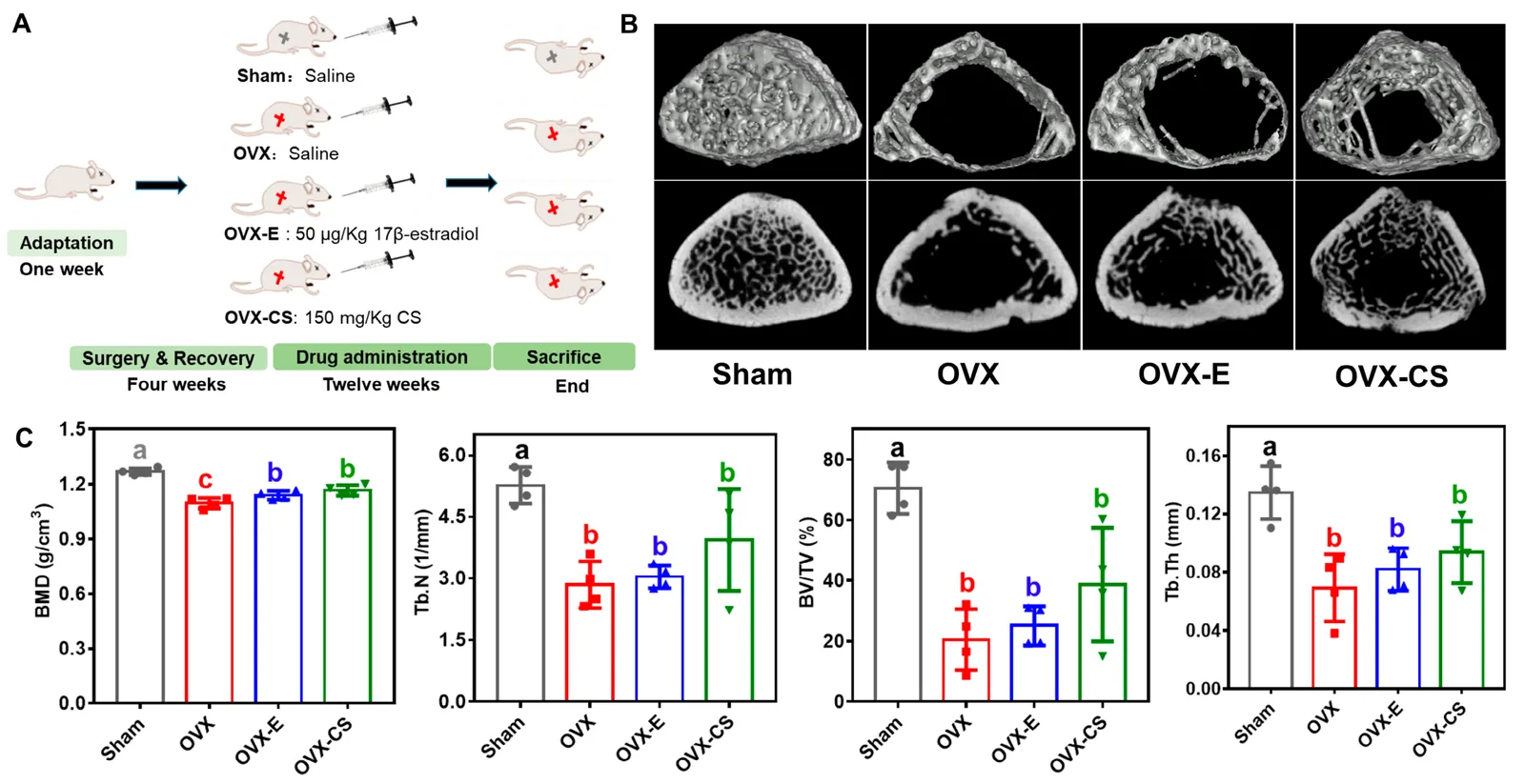
The schedule for the experiment and the osteoporosis rat model established. (**A**) The specific treatments for each group of rats. (**B**) Three-dimensional reconstruction of bone trabecula of the longitudinal section in right femurs. (**C**) Quantitative morphometric data derived from micro-CT analysis, presenting the trabecular BMD, Tb·N, BV/TV, and Tb·Th across different treatment groups. Data are presented as mean ± SD (*n* = 4 per group for micro-CT analysis). Different letters (a, b, c) indicate significant differences at *p* < 0.05, as determined by one-way ANOVA followed by Duncan’s multiple range test. Gray X denotes sham surgery (ovaries intact), whereas red X denotes ovariectomy.

The study design and treatments are shown in Figure 1A. After 12 weeks, four rats per group underwent micro-CT to verify OVX model induction. The OVX group displayed severe trabecular cavitation and disrupted bone architecture versus the Sham group (Figure 1B), confirming successful osteoporosis modeling. Both estrogen and CS interventions slightly improved trabecular appearance. BMD was significantly lower in OVX rats than in Sham, OVX-E, and OVX-CS groups (*p* < 0.05), whereas Tb·N, BV/TV, and Tb·Th showed no intergroup differences (Figure 1C). Although CS intervention seemed to improve BMD, the micro-CT scanning indicated the femoral telecentric trabecular bone connection phenotype was still cavitation without recovery compared with the Sham group. Overall, these results suggested that osteoporosis was successfully obtained by ovarian surgery, which, to some extent, was able to simulate the postmenopausal woman. CS intervention fails to effectively relieve symptoms of osteoporosis.

The decreased hormones causing the overweight problem regarding the postmenopausal women have been increasingly drawing attention. The intestinal microbiota can be altered by polysaccharides and produces various metabolites influencing the host health [25]. CS can be degraded by the gut microbiota and alter the gut microbiota composition [20,21,26] under certain conditions, which may be associated with body weight control of the host. The OVX rat was selected as a well-established model simulating postmenopausal women [27], which was able to study the issue of postmenopausal weight gain. Here, the micro-CT scanning results indicated that the OVX rat model with obvious osteoporosis symptoms established successfully can be employed to simulate the postmenopausal ones, and CS intervention fails to effectively relieve symptoms.

### 3.2. Effects of CS Supplementation on the Body Weight of OVX Rats

**Figure 2:**
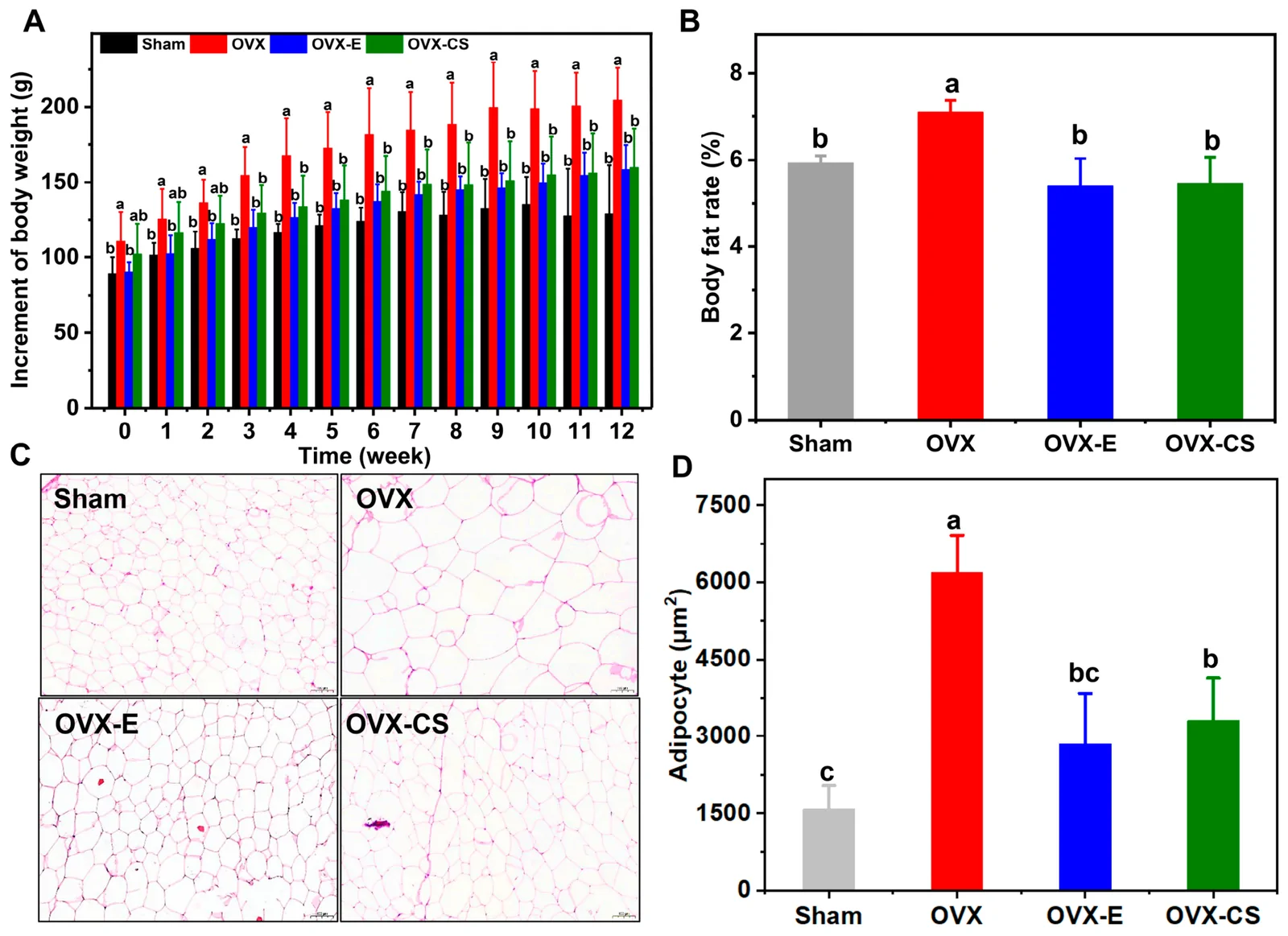
Effects of CS on the body weight of the OVX rats. (**A**) Increment of the body weight of various rat groups fed for 12 weeks. The increment of the body weight = m_1_ − m_0_ (m_1_: The determined weight of rats at the certain week; m_0_: The initial weight of rats). (**B**) The body fat rate of every group of rats. Body fat rate (%) = the weight of abdominal fat (g)/the body weight (g) × 100; (**A**,**B**) Data are presented as mean ± SD (*n* = 6 per group). (**C**) The morphology of adipocyte from the abdominal fat is assessed by H&E staining. Scale bar = 100 μm. (**D**) Quantitative analysis of adipocyte cross-sectional area (μm^2^) was performed on *n* = 3 rats per group, with 6 randomly selected fields per rat. Different letters (a, b, c) indicate significant differences at *p* < 0.05, as determined by one-way ANOVA followed by Duncan’s multiple range test.

After the rats were subjected to OVX operation, the body weight of OVX rats increased along with feeding, while the increased body weight of OVX rats supplemented with CS and estradiol was limited (Figure 2A). No difference in food intake was observed among the OVX, OVX-CS, and OVX-E groups during the 12 weeks (Figure S2). The body fat rate, assessed as the percentage of the abdominal fat in the body of the OVX group was significantly (*p* < 0.05) higher than that of the Sham group, while it was decreased in the OVX-CS and OVX-E groups (Figure 2B). The adipocyte morphology of these four groups is shown in Figure 2C. Quantitative morphometric analysis of adipocyte area is shown in Figure 2D. The mean adipocyte area in the OVX group was significantly larger than that in the Sham, OVX-E, and OVX-CS groups (*p* < 0.05). CS treatment significantly reduced adipocyte size compared with the OVX group (*p* < 0.05), although the adipocyte area did not fully recover to the Sham level. These quantitative data provide objective statistical support for the conclusion that CS counteracts estrogen-deficiency-induced adipocyte hypertrophy. Visceral indexes, including the heart, kidney, and spleen, were not different in the groups compared with the Sham group, but that of the liver in the OVX, OVX-CS, and OVX-E groups decreased remarkably compared with the Sham group (*p* < 0.05) (Figure S3).

CS intervention controlled the OVX rat body weight increase. Interestingly, compared with OVX model rats, the body weight of OVX rats subjected to the CS intervention for twelve weeks was decreased, and the food intake was not influenced by CS supplement. Recent studies indicated that high-fat diet supplemented with CS could effectively control the mice body weight gain by modulating gut microbiota [20,21]. Additionally, it was reported that CS oligosaccharide prevented body weight gain in the high-fat diet model mice by suppressing the pancreatic lipase activity and lipid accumulation [22] or maintaining the normal size of epididymal adipocytes [23]. Notably, beyond its systemic effects, high-molecular-weight CS that is not absorbed in the upper gastrointestinal tract can exert direct, localized actions. Studies have demonstrated that CS can reduce fat storage through the inhibition of small intestinal absorption of dietary fat. This effect is achieved via a dual mechanism: First, CS directly inhibits the activity of pancreatic lipase [28], the key enzyme responsible for hydrolyzing dietary triglycerides into free fatty acids and monoglycerides; second, CS inhibits the uptake of free fatty acids across the intestinal brush border membrane. Importantly, this inhibitory activity on pancreatic lipase is more pronounced for high-molecular-weight CS polysaccharides compared to their lower-molecular-weight oligosaccharide counterparts [22]. This localized action in the intestinal lumen represents a critical, direct pathway through which orally administered CS can contribute to the management of postmenopausal weight gain, complementing its systemic effects mediated by the gut microbiota.

### 3.3. Effects of CS Supplementation on the Intestinal Microbiota Composition

**Figure 3:**
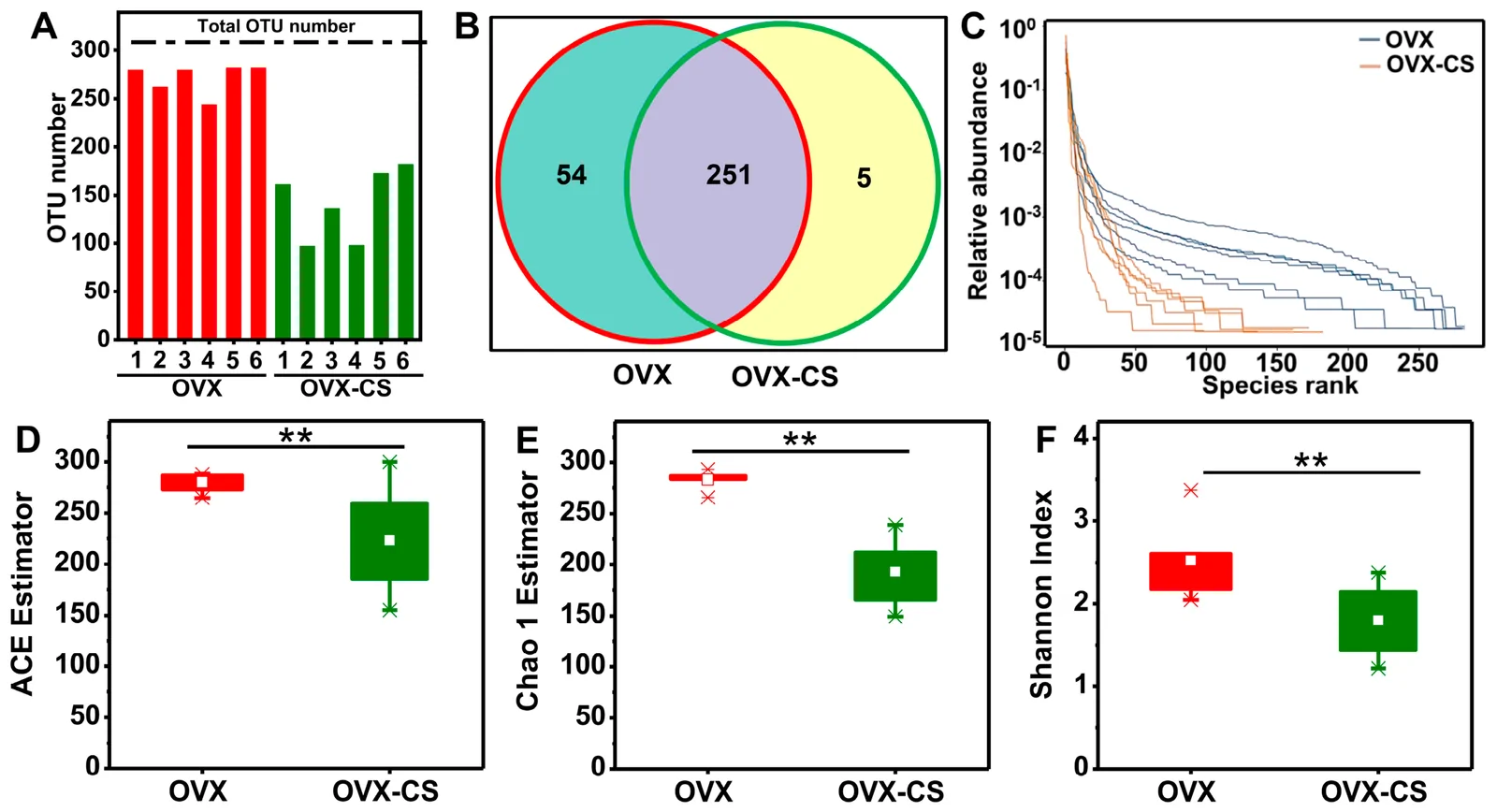
Modulation of intestinal microbial alpha diversity by CS treatment in ovariectomized rats. (**A**) Comparison of total OTU numbers between the OVX and OVX-CS groups for each biological replicate. (**B**) Venn diagram illustrating OTU overlap and group-specific OTUs in the two groups. Alpha diversity estimated by (**C**) rank abundance curve, (**D**) Ace estimator, (**E**) Chao 1 estimator, and (**F**) Shannon index. Data are presented as mean ± SD (*n* = 6 per group). ** *p* < 0.01, as determined by Student’s *t*-test (two-group comparisons).

As shown in Figure 3A,B, the total OTU counts for both the OVX and OVX-CS groups reached 310, of which 54 and 5 OTUs were uniquely detected in the OVX and OVX-CS groups, respectively. Pairwise OTU comparisons involving the OVX-E vs. OVX and Sham vs. OVX groups are available in the Supplementary Figures (Figures S4A,B and S5A,B). Alpha diversity metrics, including rank abundance curves, Ace, Chao1, and Shannon index, were used to estimate microbial richness and evenness (Figure 3C–F). The OVX-CS group showed significantly lower richness and diversity than the OVX group (*p* < 0.01), and comparable decreases were also noted in the OVX-E group (Figure S4C–F). By contrast, no substantial alterations in these indices were detected in the Sham group relative to the OVX controls (Figure S5C–F). It should be noted that the microbiota analysis in the present study was based on OTU clustering at 97% similarity, a widely accepted standard that effectively reduces sequencing noise and facilitates community-level comparisons. However, we acknowledge that emerging ASV-based methods such as DADA2 offer higher taxonomic resolution by distinguishing sequences at the single-nucleotide level. Future investigations employing ASV-based approaches may provide deeper insights into the specific microbial strains associated with the body weight-controlling effects of CS.

**Figure 4:**
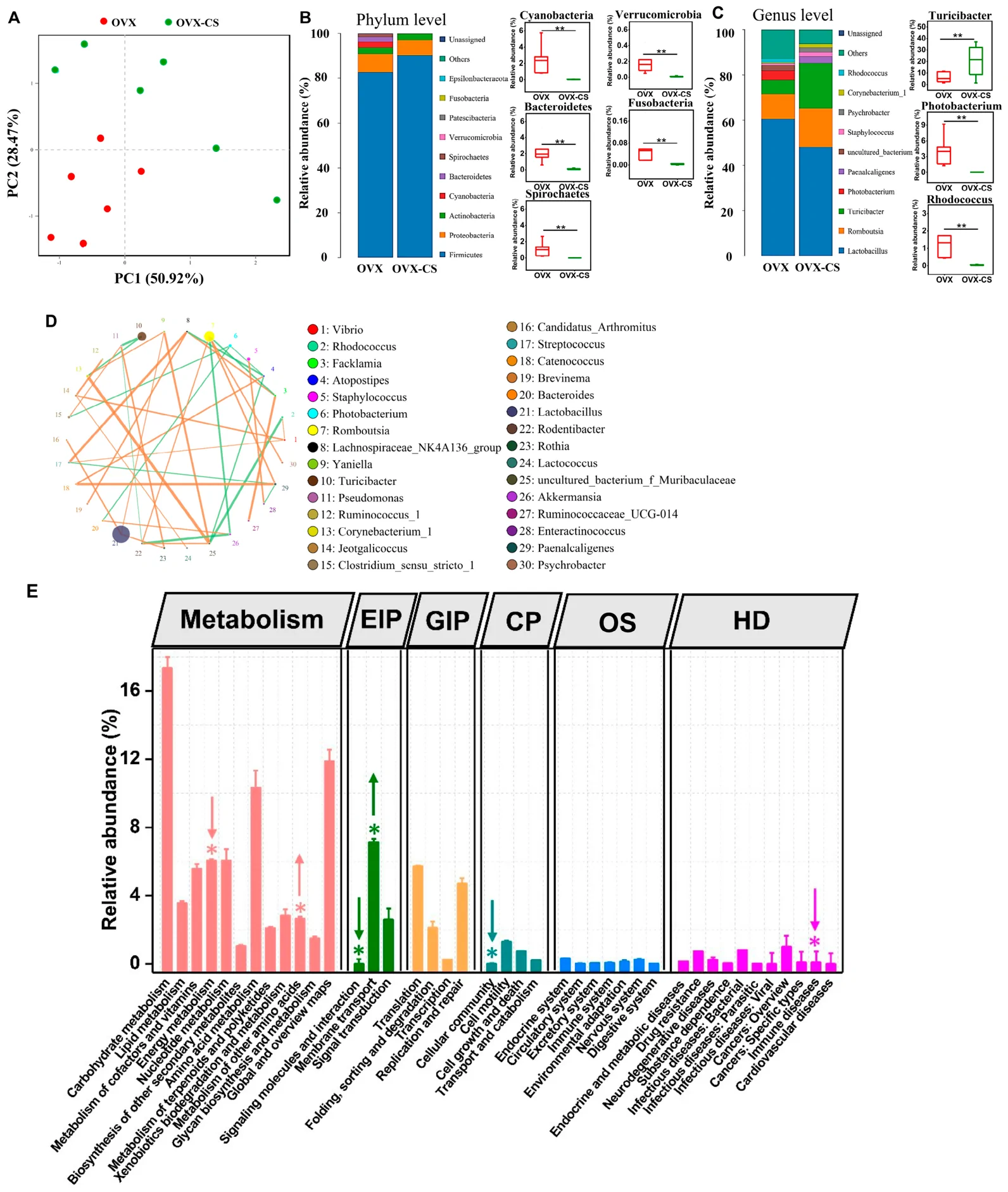
CS-induced alterations in the gut microbiota of OVX rats. (**A**) PCA ordination plot showing compositional separation between the OVX and OVX-CS groups. Taxonomic distribution of gut bacterial communities at the phylum (**B**) and genus (**C**) levels; Data are presented as mean ± SD (*n* = 6 per group for microbiota analysis). For comparisons between OVX and OVX-CS groups, ** *p* < 0.01, as determined by Student’s *t*-test. (**D**) Pearson correlation analysis of the top 30 most abundant bacteria in the gut microbiota. The size of the circle presents the relative abundance; the orange and green color of the line represent a positive and negative correlation. (**E**) KEGG-based functional prediction of the gut microbiome in OVX-CS group, with pathways grouped into six categories: Metabolism, Environmental Information Processing (EIP), Genetic Information Processing (GIP), Cellular Processes (CP), Organismal System (OS), and Human Diseases (HD). Arrows indicate significantly altered pathways relative to the OVX group (* *p* < 0.05); “↑”/”↓” denote up-regulation/down-regulation, respectively.

PCA of the gut microbiota profiles demonstrated distinct clustering between the OVX and OVX-CS groups, with PC1 and PC2 together explaining 79.39% of the overall variance, suggesting good model stability and reliability (Figure 4A). Similar PCA results were obtained for the comparisons involving OVX vs. OVX-E and OVX vs. Sham, both yielding a cumulative variance explanation of 76.99% (Figure S6A,B). At the phylum level, *Firmicutes*, *Proteobacteria*, *Actinobacteria*, *Cyanobacteria*, *Bacteroidetes*, *Spirochaetes*, *Verrucomicrobia*, *Patescibacteria*, *Fusobacteria* and *Epsilonbacteraeota* were determined, and the dominant phylum in the OVX and OVX-CS groups were *Firmicutes* and *Proteobacteria* (Figure 4B). At the genus level, *Lactobacillus*, *Romboutsia*, *Turicibacter*, *Photobacterium*, *Paenalcaligenes*, *Staphylococcus*, *Psychrobacter*, *Corynebacterium* and *Rhodococcus* were detected (Figure 4C). The proportional bar graphs showed the relative abundance of the microbiota at the phylum and genus levels, and the box plots presented a significant (*p* < 0.01) changed microbiota in the OVX and OVX-CS groups. After OVX rats supplemented with CS for 12 weeks, the relative abundance of *Turicibacter* increased significantly while *Photobacterium* and *Rhodococcus* decreased obviously (*p* < 0.01). For the OVX vs. OVX-E groups and the OVX vs. Sham groups, the microbiota determined at the phylum level was the same as OVX vs. OVX-CS, while at the genus level, specific significant changes were different. At the phylum level, compared with the OVX group, the relative abundance of *Cyanobacteria*, *Bacteroidetes* and *Spirochaetes* from the OVX-E group decreased significantly (*p* < 0.01), while there was no obvious change in the Sham group (Figure S6C). At the genus level, compared with the OVX group, the relative abundance of *Photobacterium*, *Vibrio* and *Brevinema* from the OVX-E group decreased significantly (*p* < 0.01), and *Turicibacter* and *Romboutsia* were increased while *Lactobacillus* was decreased in the Sham group (Figure S6D). Furthermore, we examined the correlation patterns among the 30 most abundant bacterial genera (Figure 4D). A number of significant pairwise correlations were identified: *Lactobacillus* was positively associated with *Yaniella*, *Candidatus_Arthromitus*, and *Rothia*, while *Turicibacter* exhibited inverse relationships with *Pseudomonas* and *Corynebacterium_1*. Negative correlations were also observed between *Photobacterium* and three taxa—*Atopostipes*, *Bacteroides*, and *Clostridium_sensu_stricto_1*. As for *Rhodococcus*, it showed a negative correlation with *Uncultured_bacterium_f_Muribaculaceac* and a positive one with *Vibrio*.

It is important to acknowledge that certain genera detected in our study, particularly *Photobacterium* and *Vibrio*, are more commonly associated with marine environments and fish intestinal microbiota, rather than being typical residents of the mammalian gut. While *Vibrio* has been documented in rat intestinal microbiota in several published studies [29,30], the presence of *Photobacterium* warrants particular caution. Several factors may contribute to the detection of these taxa in our samples. First, dietary or environmental sources, such as components of the standard chow diet, could introduce environmental bacteria into the gastrointestinal tract. Second, low-abundance taxa are more susceptible to misclassification, as approximately 60% of bacterial genera in the rat ileum are exclusive and present at low abundance [31]. Third, reagent or laboratory contamination is a well-recognized confounder in 16S rRNA sequencing studies, particularly for low-biomass samples [32]. Importantly, our primary conclusions are based on the differential abundance of these genera between the OVX and OVX-CS groups, rather than their absolute presence. The consistent and statistically significant differences observed (*p* < 0.01), together with coordinated changes in other taxa and their correlation with specific metabolite profiles, support the biological relevance of the observed microbiota shifts. Nevertheless, future studies incorporating negative sequencing controls and ASV-based methods (e.g., DADA2) would help to further distinguish true biological signals from potential contaminants.

Functional profiling of the bacterial community in the OVX-CS group was performed using KEGG database annotation (Figure 4E). The prediction yielded 41 KEGG orthologs, which were assigned to six broad functional classes: metabolism, EIP, GIP, CP, OS, and HD. Metabolism-related functions predominated, with the major subcategories being carbohydrate metabolism, amino acid metabolism, global and overview maps, and energy metabolism. Furthermore, comparative analysis between the OVX-CS and OVX groups revealed significant differences in several specific pathways, including energy metabolism, other amino acid metabolism, signaling molecules and interaction, membrane transport, cellular community, and immune diseases.

The gut microbiota composition of OVX rats was notably affected by CS treatment. Although the oral bioavailability of CS is limited (less than 20%) [18,33], and its absorption predominantly occurs in the form of small oligosaccharides derived from microbial breakdown [34], pointing to a reciprocal interplay between CS and the gut microbiome. Increasing studies showed CS could modify the gut microbiota structure [20,21,22,35]. Additionally, increasing research suggested that CS can improve some diseases such as obesity [20,21], rheumatoid arthritis [36] and colorectal cancer [35], by regulating the gut microbiota. It was suggested that the functions of CS can be shown by the interplaying with the gut microbiota. In our study, CS supplement was associated with the alterations in gut microbiota at the phylum and genus level as well. Fucosylated CS decreased the abundance of *Bacteroidetes* of the obese mice [37], which was consistent with our results. *Verrucomicrobia* and *Bacteroidetes* could hydrolyze CS at high rates in the water column [38]; the communities in this study, however, decreased in the OVX-CS group, which probably ascribes the microbiota complexity in vivo. In addition, little research was found regarding the interaction of CS and the communities of *Spirochaetes*, *Cyanobacteria* and *Fusobacteria*. And the specific communities of *Turicibacter*, *Photobacterium* and *Rhodococcus* unreported in other literature may be the new bacterial communities in the OVX rat induced by CS to control the body weight, which needs to be confirmed in future work.

Research suggests that the interaction of estrogen and gut microbiota existed, and the estrogen metabolism can be influenced by gut microbiota [39,40,41]. Based on isotopic tracing, approximately 10–15% of estrogens are excreted in the feces in their conjugated state, and once in the gut, these conjugates serve as substrates for bacterial enzymes, particularly β-glucuronidase and β-galactosidase, which cleave the conjugating moieties and release free estrogens that can be reabsorbed into the bloodstream [42,43]. Interestingly, we found CS intervention decreased the β-estradiol-17β-glucuronide (Table S1, Negative, M_1718) in the intestine. It was speculated that CS probably inhibited the estrogen conjugation by influencing the intestinal microbiota, which promoted estrogen reabsorption. Our finding that ovariectomy shifted the relative abundances of Bacteroidetes and Firmicutes in adult rats is corroborated by previous work [44]. Estrogen deficiency is known to perturb the homeostatic interaction between the gut microbiota and circulating estrogens; under such conditions, the microbial–host endocrine axis is re-established to adapt to the altered physiological state [11]. These results probably imply that CS intervention may modulate gut microbiota to reduce estrogen conjugation and promote reabsorption. However, the potential specific interaction of gut microbiota influenced by CS intervention and estrogen should be further investigated.

### 3.4. Effects of CS Supplementation on Metabolite Profiles

**Figure 5:**
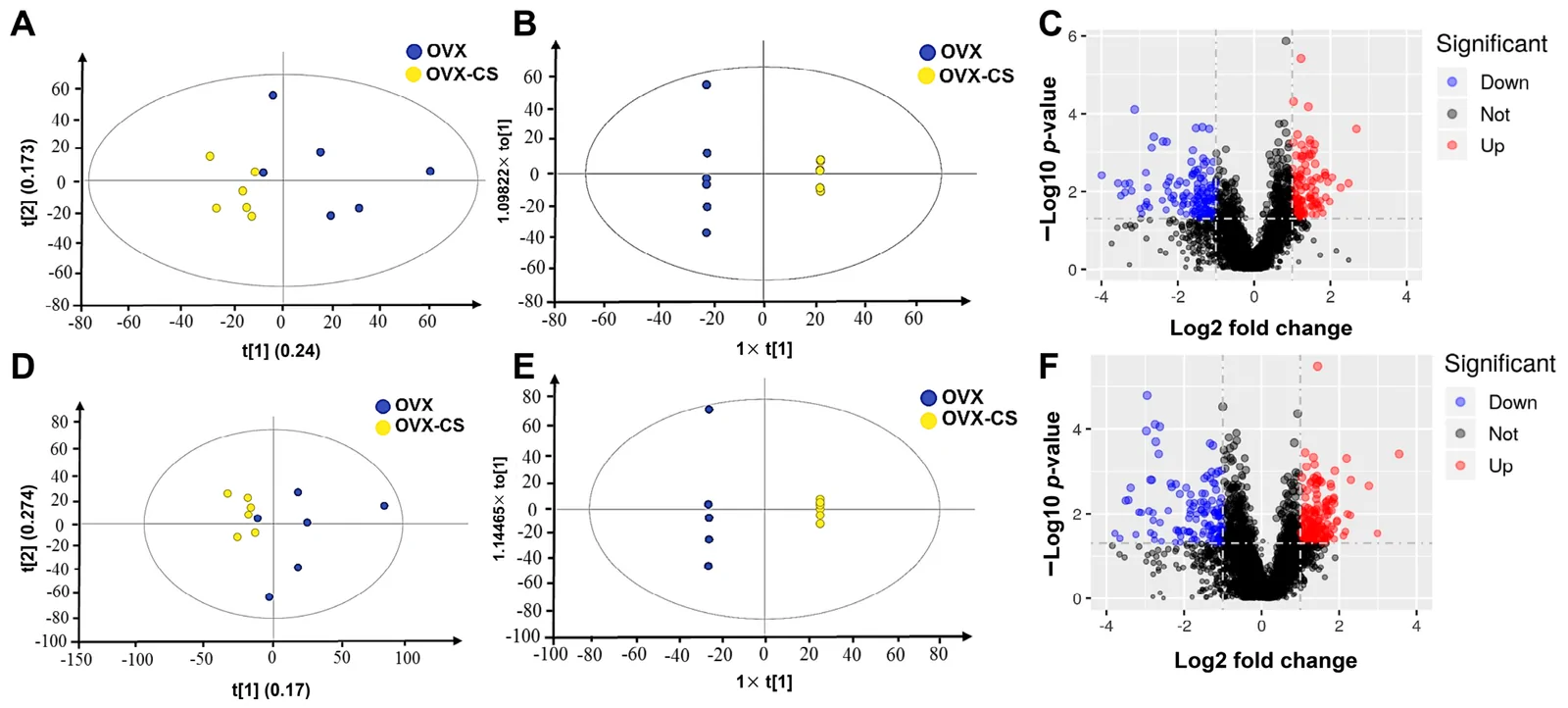
CS-induced alterations in the intestinal metabolome of OVX rats. Negative-ionization mode data: PCA (**A**) and OPLS-DA (**B**) score plots showing group separation between OVX and OVX-CS rats, and volcano plot (**C**) displaying differentially abundant metabolites. Corresponding positive-ionization mode data: PCA (**D**), OPLS-DA (**E**), and volcano plot (**F**). In the volcano plots (**C**,**F**), red and blue dots represent metabolites with significantly elevated or reduced abundance, respectively; black dots indicate no significant difference; dot size is proportional to the VIP value obtained from OPLS-DA. PCA and OPLS DA score scatter plots were generated from metabolomics data of *n* = 6 rats per group (OVX and OVX-CS).

Intestinal metabolomic profiling was performed on OVX-CS and OVX rats to evaluate the global metabolic effects of CS-supplemented diets. PCA was applied to visualize data distribution and group clustering. The resulting score plots for the negative- and positive-ionization modes are shown in Figure 5A and Figure 5D, respectively. After interquartile range-based noise filtering and exclusion of unmatched features, 262 metabolites (VIP > 1, *p* < 0.05) were identified from the positive- and negative-ion mode data (Table S1). Unsupervised PCA already revealed a clear separation between the OVX-CS and OVX groups in both ionization modes. To further enhance group discrimination and elucidate the key variables responsible for the observed clustering, OPLS-DA was applied (Figure 5B,E). The differential metabolites identified across the two modes are summarized as volcano plots in Figure 5C,F. Compared with the OVX group, the changed metabolites included carbohydrate, fatty acid, peptide, carboxylic acid and their conjugates, or other compounds. They changed (up- or down-regulating) significantly in the OVX-CS group. Particularly, the carbohydrates, fatty acids and conjugates increased dramatically, which included α, α-trehalose (Negative, M_269), N-Acetyl-β-D-galactosamine (Negative, M_626), gluconic acid (Negative, M_36), 9-KODE (Positive, M_1089), oleic acid (Negative, M_3), 12, 13-diHOME (Negative, M_209), and 9(Z),11(E)-conjugated linoleic acid (Negative, M_2). Most of the peptides decreased significantly. Additionally, the short-chain fatty acid such as valeric acid decreased.

### 3.5. Metabolite Enrichment and the Correlation Analysis with the Intestinal Microbiota

**Figure 6:**
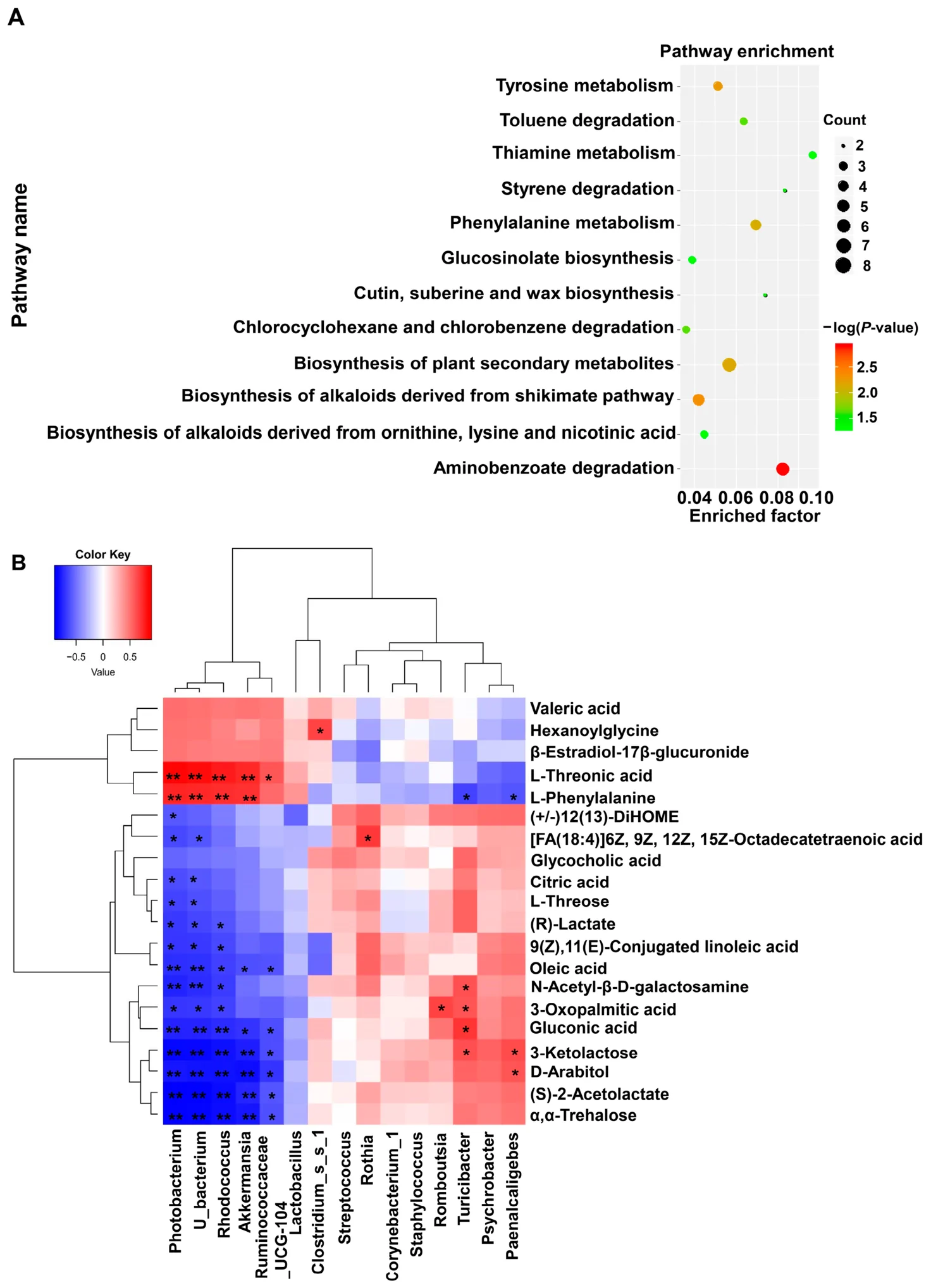
Integration of metabolomic enrichment and microbiota–metabolite correlation. (**A**) KEGG pathway enrichment analysis comparing the OVX-CS and OVX groups (*n* = 6 rats per group). (**B**) Pearson’s correlation of interesting metabolites and intestinal microbial at genus (OVX-CS group and OVX group). U_bacterium: Unculture_bacterium; Clostridium_s_s_1: Clostridium_sensu_stricto_1. * *p* < 0.05, ** *p* < 0.01, as determined by Pearson’s correlation test (two-tailed *t*-test).

KEGG enrichment of differential metabolites identified five significantly (*p* < 0.05) affected pathways: phenylalanine metabolism, glucosinolate biosynthesis, thiamine metabolism, tyrosine metabolism, and aminobenzoate degradation (Figure 6A), linking to L-phenylalanine, citric acid, benzoic acid, and oleic acid. Pearson’s correlation between selected genera and differential metabolites (OVX-CS vs. OVX) is shown as a heatmap (Figure 6B). Overall, 16, 15, 12, 8 and 7 metabolites exhibited remarkable correlation with *Photobacterium*, *Unculture_bacterium*, *Rhodococcus*, *Akkermansia* and *Ruminococcaceae_UCG-104*. *Turicibacter* exhibited a significant (*p* < 0.05) correlation with five altered metabolites, and *Paenalcaligebes* was correlated with three metabolites. Additionally, *Clostridium_sensu_stricto_1*, *Rothia* and *Romboutsia* were significantly (*p* < 0.05) correlated with hexanoylglycine, [FA (18:4)] 6Z, 9Z, 12Z, 15Z-Octadecatetraenoic acid and 3-oxopalmitic acid, respectively.

CS-induced shifts in the gut microbiota of OVX rats were parallel with significant changes in the intestinal metabolome. Notably, α, α-trehalose emerged as the most profoundly altered carbohydrate in the OVX-CS group. The metabolic benefits of trehalose have been documented in multiple contexts: it counteracts diet-induced obesity, alleviates hepatic steatosis by reducing lipid accumulation and enhancing glycogen storage [45,46], and suppresses hepatic endoplasmic reticulum stress and inflammatory responses in aging models [47]. Nevertheless, the therapeutic efficacy of oral trehalose remains controversial, as one recent study failed to demonstrate protective effects against obesity and insulin resistance [48]. Here, trehalose showed a negative correlation with the bacterial communities of *Photobacterium*, *Unculture bacterium*, *Rhodococcus*, *Akkermansia* and *Ruminococcaceae_UCG-104* (*p* < 0.01). Another up-regulated carbohydrate metabolite was N-acetyl-β-D-galactosamine positively correlated with *Turicibacter*, which might be from the metabolism of CS directly because it consisted of CS. Conjugated linoleic acid has beneficial effects on body composition and weight loss in humans and animals by reducing the size of fat cells, altering the evolution of adipocytes and regulating lipolytic pathways [49,50,51,52,53]. 12,13-diHOME, a brown adipose tissue-derived lipid, is inversely associated with adiposity, and exogenous 12,13-diHOME administration has been shown to decrease circulating triglycerides and improve lipid tolerance in obese mouse models [54]. Here, 9(Z),11(E)-conjugated linoleic acid and 12,13-diHOME were up-regulated in the OVX-CS group, and the conjugated linoleic acid exhibited a negative correlation with *Photobacterium* and *Rhodococcus*, and 12,13-diHOME showed a negative correlation with *Photobacterium*. Therefore, we speculated that CS probably altered the abundances of *Turicibacter*, *Photobacterium* and *Rhodococcus* and promoted the production of some metabolites including α, α-trehalose, conjugated linoleic acid, 12,13-diHOME and other metabolites, which ultimately influenced the body weight.

**Figure 7:**
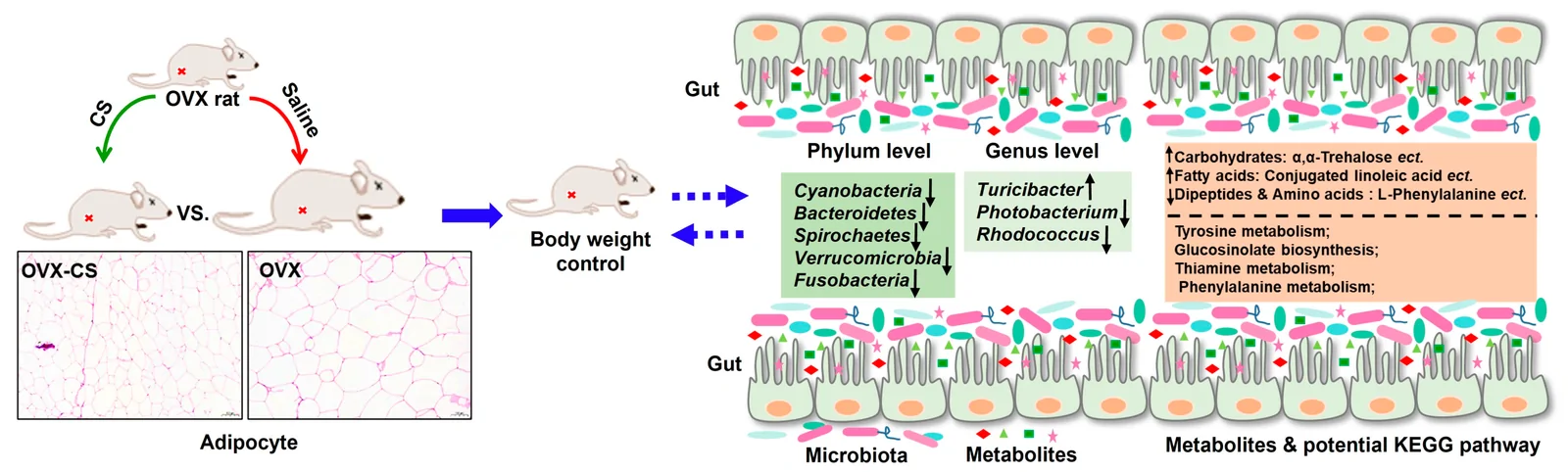
A working model summarizing the hypothesized mechanisms by which CS supplementation influences gut microbiota–metabolite–host interactions to control body weight in OVX rats. Dashed arrows denote the proposed pathways and associations underlying the anti-obesity effects of CS.

Beyond the microbiota-dependent mechanism discussed above, an intriguing and complementary hypothesis merits consideration. Given that CS is a polyanionic polysaccharide capable of interacting with amphiphilic and hydrophobic molecules [55], it is plausible that orally administered CS could physically associate with dietary lipids and estrogens (or their conjugates) within the intestinal lumen. This could lead to the formation of supramolecular complexes or nanoparticle-like structures, as has been demonstrated for CS-based oil-core nanocapsules [56]. Hydrophobically modified CS has also been shown to self-assemble into micelles, and its conjugation with fatty acids (e.g., α-linolenic acid) significantly enhances intestinal absorption through transient tight-junction opening [57]. Such nanostructures might facilitate the intestinal absorption of the incorporated estrogen via transcellular or lymphatic routes. The lymphatic pathway is particularly intriguing, as it is well established that lipid-based nanoparticles can transport lipophilic cargo from the intestinal lumen to the mesenteric lymphatics, thereby bypassing first-pass hepatic metabolism [58]. While the above hypothesis requires direct experimental validation, it offers a novel, physically plausible explanation for how CS supplementation may mitigate body weight gain associated with estrogen deficiency in OVX rats, thereby complementing the established gut microbiota-dependent pathway. Overall, due to the lack of direct experimental evidence, the proposed involvement of the gut microbiota–metabolite–host axis remains speculative and hypothesis-generating as shown in Figure 7, rather than a demonstrated mechanistic pathway.

## 4. Conclusions

Our results demonstrated that CS supplementation can alleviate the body weight increase in ovariectomized rats, accompanied by modifications in gut microbiota composition and metabolite profiles. These findings provide a plausible explanation for the potential role of CS as a functional food component against body weight gain. However, it should be emphasized that these results are derived from an animal model and cannot be directly extrapolated to humans without further validation. While the OVX rat is a widely accepted preclinical model for postmenopausal physiology, it does not fully recapitulate human menopause, as surgical ovariectomy induces an acute and complete loss of ovarian hormones, whereas human menopause is a gradual process occurring over several years. Moreover, although the CS dose (150 mg/kg per day) used in this study translates to a human equivalent dose, the efficacy observed in rats requires validation in well-designed human trials before any translational recommendations can be made for postmenopausal populations. Our data are derived from correlational analyses (16S rRNA sequencing and untargeted metabolomics), and the observed associations between specific bacterial taxa, metabolites, and host phenotypes do not, by themselves, demonstrate causality. While our findings are consistent with the hypothesis that CS modulates body weight through the gut microbiota–metabolite axis, alternative explanations, such as CS directly affecting host metabolism or the observed microbial/metabolic changes being secondary to the body weight phenotype, cannot be excluded.

Several limitations should be acknowledged. The cause-effect relationships among CS intervention, gut microbiota remodeling, metabolite alterations, and body weight control require further investigation through functional studies, including fecal microbiota transplantation (FMT) in germ-free rats, targeted metabolite supplementation, and microbiota depletion experiments. Additionally, the metabolomic findings were based on putatively annotated compounds without confirmation using authentic reference standards; future targeted validation with reference standards and stable isotope-labeled internal standards would enhance both the quantitative and qualitative confidence in metabolite identification. Furthermore, the potential influence of CS sulfation patterns and the roles of other glycosaminoglycans merit systematic investigation. Collectively, while our multi-omics approach has generated robust correlational associations, these findings are hypothesis-generating rather than hypothesis-confirming, and causal validation is essential before any mechanistic claims can be made.

## Data Availability

The data that support the findings of this study are available from the authors upon reasonable request.

## Supplementary Materials

The following supporting information can be downloaded at: https://www.mdpi.com/article/10.3390/biom16081095/s1, Figure S1: The information of CS in this study.; Figure S2: The food intake of every group rats during the 12 weeks; Figure S3: The visceral index of the heart, kidney, liver and spleen from the different group rats; Figure S4: Changes of global gut microbiota after intervention in OVX and OVX-E groups; Figure S5: Changes of global gut microbiota after intervention in OVX and Sham groups; Figure S6: Effect of estradiol on the gut microbiota; Table S1: The information of significantly changed metabolites (262) from OVS-CS group vs. OVX group.
