# Supplementary material for: Chondroitin Sulfate Supplementation Is Associated with Body Weight Control in Ovariectomized Rats: A Multi-Omics Study of Gut Microbiota and Metabolite Profiles
Source: Biomolecules. 2026 Jul 27;16(8):1095. doi: 10.3390/biom16081095 (PMC13509910; doi:10.3390/biom16081095)
Supplement: Supplementary file 1 [file biomolecules-16-01095-s001.zip › biomolecules-4426323-supplementary.pdf]

Chondroitin Sulfate Supplementation Is Associated with Body Weight Control in Ovariectomized Rats: A Multi-Omics Study of Gut Microbiota and Metabolite Profiles

Qingshan Shen[1,2], Yanli Ma[1,2], Chunhui Zhang[3], Yujie Guo[3], Xiaojie Qin[3*]

[1]*Henan Key Laboratory of Zhang Zhongjing Formulae and Herbs for Immunoregulation, Zhang Zhongjing College of Chinese Medicine, Nanyang Institute of Technology, Changjiang Road 80, Nanyang, 473004, China.*

[2]*School of Food and Health, Henan College of Traditional Chinese Medicine, Hansheng Road 1222, Nanyang, 473013, China.*

[3]*Key Laboratory of Agro-Products Processing, Ministry of Agriculture and Rural Affairs, Institute of Food Science and Technology, Chinese Academy of Agricultural Sciences, Beijing, 100193, China*

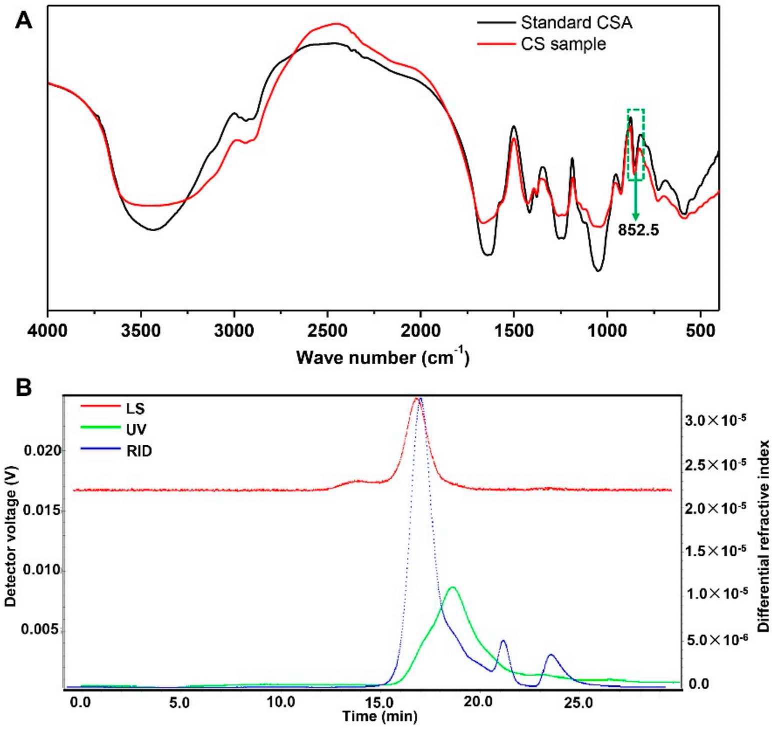

Figure S1. The information of CS in this study. (A) The FTIR spectra of standard type A chondroitin sulfate (CSA) and CS sample. For CS, the absorption peak at 852 cm^{-1} is recognize as the characteristic absorption of CSA. (B) The CS sample chromatograms obtained by gel permeation chromatography with a multi-angle laser light scattering system were used to assess the molecular weight that is 16.37 kDa.

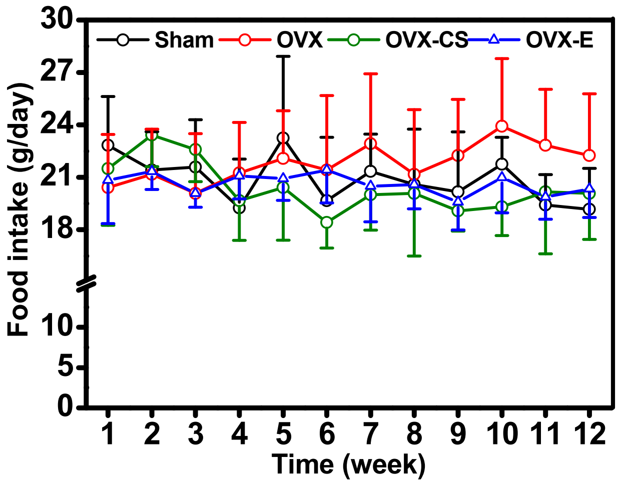

Figure S2. The food intake of every group rats during the 12 weeks. The amount of food intake is evaluated randomly on a certain day of every week, which is used as the food intake every day in this week. No significant difference (P>0.05, n=6) was observed in OVX, OVX-CS and OVX-E groups during the 12 weeks.

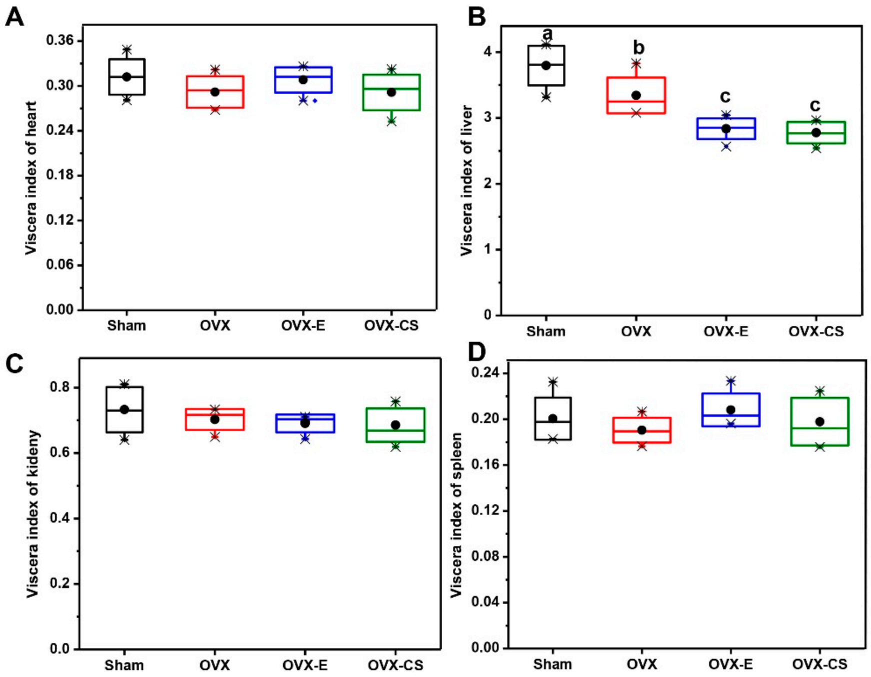

Figure S3. The visceral index of the heart, kidney, liver and spleen from the different group rats. Visceral index (%) = the weight of organ (g)/the body weight (g) × 100. The different letters indicate a significant difference (P<0.05, n=6).

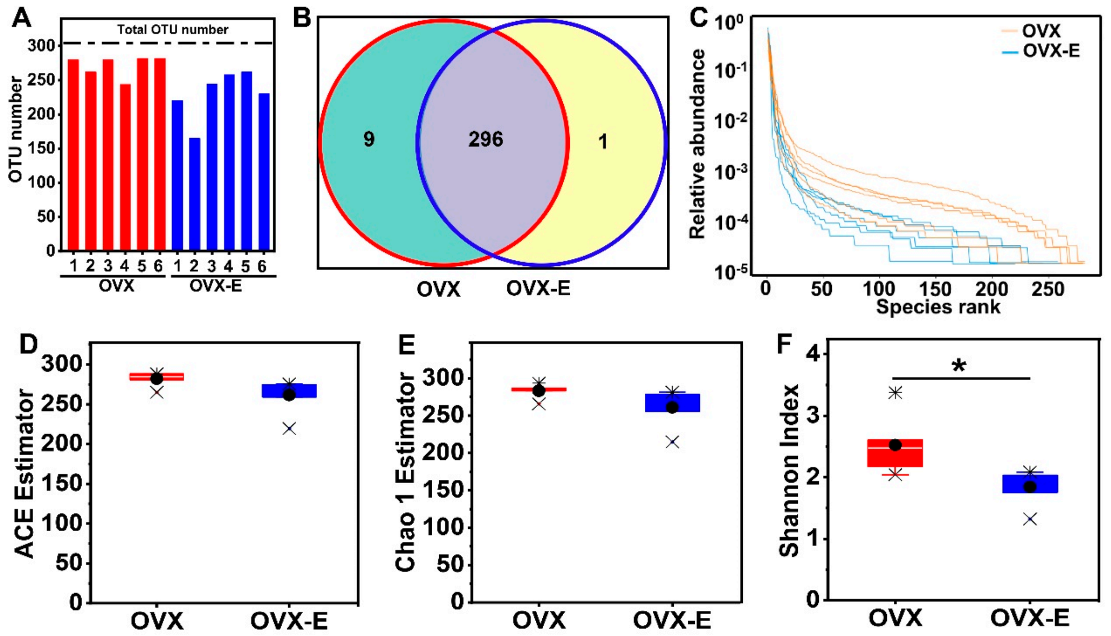

Figure S4. Changes of global gut microbiota after intervention in OVX and OVX-E groups. (A) The OTU number of the microbiota from each sample from the OVX and OVX-E group. (B)The venn plot for OTUs of the OVX and OVX-E group. α-Diversity estimated by (C) rank abundance curve (D) Ace estimator, (E) Chao 1 estimator and (F) Shannon index. *P<0.05, n=6.

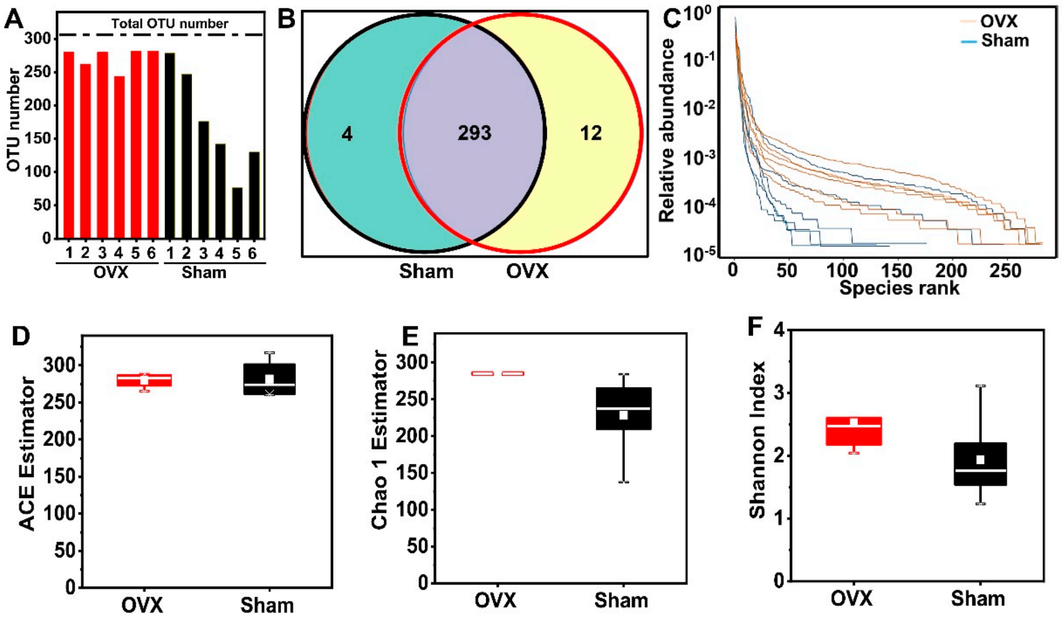

Figure S5. Changes of global gut microbiota after intervention in OVX and Sham groups. (A) The OTU number of the microbiota from each sample from the OVX and Sham group. (B)The venn plot for OTUs of the Sham and OVX group. α-Diversity estimated by (C) rank abundance curve (D) Ace estimator, (E) Chao 1 estimator and (F) Shannon index.

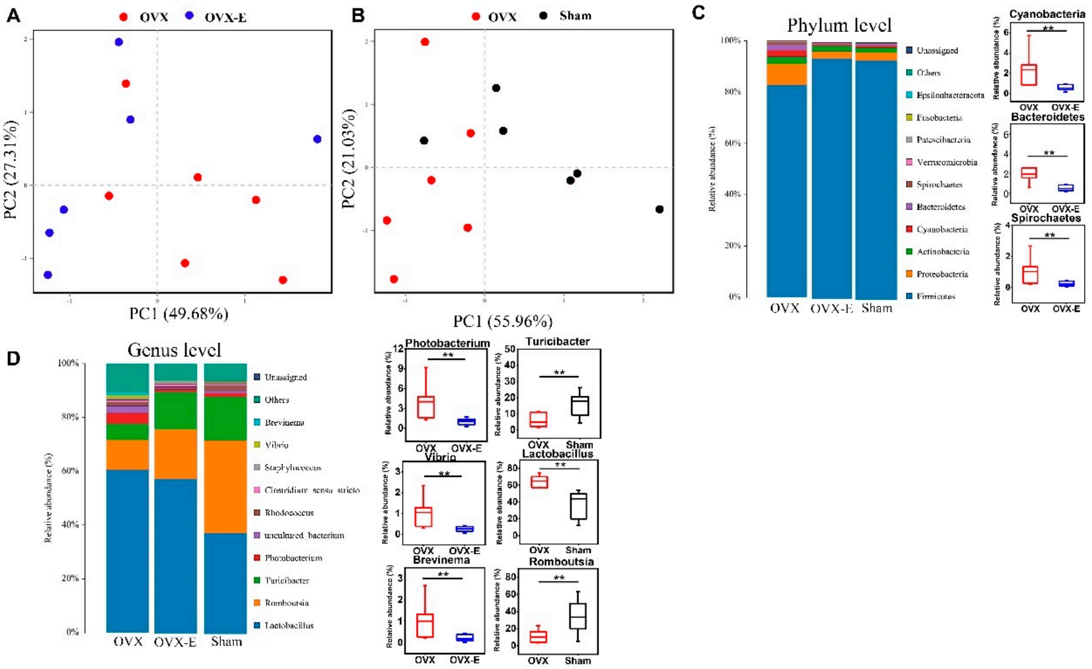

Figure S6. Effect of estradiol on the gut microbiota. (A, B) PCA shows the separation of the gut microbiota composition of the Sham or OVX rats with or without estradiol. Relative abundance of the microbiota at phylum level (C) and genus level (D). $**P < 0.01$, n=6.

Table S1. The information of significantly changed metabolites (262) from OVS-CS group vs. OVX group

Model	ID	Name	Formula	MW	VIP	*P*-value	FC
Negative	M_2	9(Z),11(E)-Conjugated linoleic acid	$C_{18} H_{32} O_2$	280.23965	1.40618	0.0076344	1.5033543
Negative	M_3	Oleic acid	$C_{18} H_{34} O_2$	282.25531	1.45182	0.0032349	1.7088242
Negative	M_24	L-Phenylalanine	$C_9 H_{11} N O_2$	165.07774	1.40315	0.0138648	0.6887101
Negative	M_36	Gluconic acid	$C_6 H_{12} O_7$	196.05728	1.38345	0.0120052	1.5866171
Negative	M_45	Pinolenic acid	$C_{18} H_{30} O_2$	278.224	1.63606	0.0003093	1.7881016
Negative	M_65	2-Oxooctadecanoicacid	$C_{18} H_{34} O_3$	298.2502	1.55129	0.0005494	1.5568973
Negative	M_75	5-Aminovaleric acid	$C_5 H_{11} N O_2$	117.07757	1.43061	0.0069715	0.5989775
Negative	M_77	Glycocholic acid	$C_{26} H_{43} N O_6$	465.30806	1.21324	0.0385868	2.8756877
Negative	M_100	3,4-Dihydroxybenzenesulfonic acid	$C_6 H_6 O_5 S$	189.99257	1.41326	0.0095836	0.1080705
Negative	M_110	Valeric acid	$C_5 H_{10} O_2$	102.06657	1.16431	0.0411049	0.4305956
Negative	M_153	(-)-Quebrachitol	$C_7 H_{14} O_6$	194.07825	1.4266	0.0192952	1.8428536
Negative	M_154	Uracil	$C_4 H_4 N_2 O_2$	112.02582	1.38905	0.0062747	0.5663479
Negative	M_181	4-Oxoproline	$C_5 H_7 N O_3$	83.03572	1.19993	0.0281079	0.7862547
Negative	M_207	(9Z)-(13S)-12_13-Epoxyoctadeca-9_11-dienoicacid	$C_{18} H_{30} O_3$	294.2191	1.25929	0.0410303	1.4257174
Negative	M_209	(+/-)12(13)-DiHOME	$C_{18} H_{34} O_4$	296.2346	1.30472	0.0146548	1.6158852
Negative	M_214	D-Raffinose	$C_{18} H_{32} O_{16}$	504.16834	1.3807	0.0235017	2.8591528
Negative	M_215	(R)-10-Hydroxystearate	$C_{18} H_{36} O_3$	300.26586	1.24393	0.047149	1.6011944
Negative	M_238	2-(beta-D-Glucosyl)-sn-glycerol	$C_9 H_{18} O_8$	254.09966	1.373	0.0299683	1.8485594
Negative	M_248	trans-Cinnamic acid	$C_9 H_8 O_2$	148.05118	1.4342	0.0062193	0.6686636
Negative	M_259	lys-leu	$C_{12} H_{25} N_3 O_3$	259.18913	1.25373	0.0375595	0.5787878
Negative	M_266	L-Threonic acid	$C_4 H_8 O_5$	136.03721	1.6458	7.82E-05	0.1142491
Negative	M_269	α,α-Trehalose	$C_{12} H_{22} O_{11}$	342.11567	1.63091	0.00062	3.0119037
Negative	M_312	δ-Gluconic acid δ-lactone	$C_6 H_{10} O_6$	178.04663	1.31224	0.0389943	1.4417308

Negative	M_318	Citric acid	$C_6 H_8 O_7$	192.02605	1.28117	0.0214714	2.2219932
Negative	M_324	Glycyl-L-leucine	$C_8 H_{16} N_2 O_3$	188.11507	1.29478	0.0213792	0.5651149
Negative	M_329	Gly-Phe	$C_{11} H_{14} N_2 O_3$	222.0996	1.56909	0.0002479	0.4425895
Negative	M_353	hydroprene	$C_{17} H_{30} O_2$	266.22412	1.15514	0.0349779	0.3786936
Negative	M_371	acrovestone	$C_{32} H_{42} O_8$	554.29036	1.37287	0.0130883	0.3603492
Negative	M_373	16-Hydroxyhexadecanoic acid	$C_{16} H_{32} O_3$	272.23477	1.37263	0.0180202	1.4853742
Negative	M_399	(1S,2S,3R,4S,5R,6S)-2,3,4,6-Tetrahydroxy-5-methoxycyclohexyl alpha-D-galactopyranoside	$C_{13} H_{24} O_{11}$	356.13141	1.5085	0.0077829	2.8287831
Negative	M_407	[FA(18:3)]13S-hydroperoxy-9Z_11E_14Z-octadecatrienoicacid	$C_{18} H_{30} O_4$	310.21391	1.49835	0.0092906	1.9262537
Negative	M_440	2_7-Anhydro-alpha-N-acetylneuraminicacid	$C_{11} H_{17} N O_8$	291.09498	1.43418	0.018002	2.3810633
Negative	M_469	(2S_3R)-3-Hydroxybutane-1_2_3-tricarboxylate	$C_7 H_{10} O_7$	206.04172	1.4072	0.0092755	1.9861848
Negative	M_498	2(alpha-D-Mannosyl)-D-glycerate	$C_9 H_{16} O_9$	268.07874	1.42773	0.0160854	2.0315327
Negative	M_514	5-Hydroxytryptophan	$C_{11} H_{12} N_2 O_3$	220.08726	1.3561	0.0125686	0.2320263
Negative	M_527	HEPES	$C_8 H_{18} N_2 O_4 S$	238.09802	1.38126	0.0128476	0.2749167
Negative	M_531	GLY-MET	$C_7 H_{14} N_2 O_3 S$	206.07151	1.37409	0.016856	0.3504708
Negative	M_555	Phenylacetaldehyde	$C_8 H_8 O$	120.05636	1.24155	0.0214654	0.364342
Negative	M_561	3-(3_4-Dihydroxypyridin-1-yl)-L-alanine	$C_8 H_{12} N_2 O_4$	200.07896	1.28763	0.026143	1.8176284
Negative	M_562	2-Hydroxyphenylalanine	$C_9 H_{11} N O_3$	181.07279	1.40396	0.010299	0.6011007
Negative	M_575	3-Phenyllactic acid	$C_9 H_{10} O_3$	166.06184	1.24726	0.0200517	0.35525
Negative	M_582	DL-4-Hydroxyphenyllactic acid	$C_9 H_{10} O_4$	182.05676	1.28396	0.0167839	0.1890873
Negative	M_596	(2R)-1-{[(2-Aminoethoxy)(hydroxy)phosphoryl]oxy}-3-hydroxy-2-propanyl (7Z,10Z,13Z,16Z)-7,10,13,16-docosatetraenoate	$C_{27} H_{48} N O_7 P$	529.31579	1.15055	0.0390531	0.6116352
Negative	M_626	N-Acetyl-beta-D-galactosamine	$C_8 H_{15} N O_6$	221.08912	1.52206	0.0010069	1.4917425

Negative	M_634	N2-Acetyl-L-aminoadipate	$C_8 H_{13} N O_5$	203.07864	1.30869	0.0459722	1.5501162
Negative	M_687	AMPA	$C_7 H_{10} N_2 O_4$	186.06318	1.28067	0.0203681	1.4506037
Negative	M_696	L-Threose	$C_4 H_8 O_4$	120.04192	1.38219	0.0043724	1.509298
Negative	M_714	3-Hydroxypropenoate	$C_3 H_4 O_3$	88.0156	1.48103	0.0024335	1.7893307
Negative	M_729	[FAmethyl(18:0)]11R_12S-methylene-octadecanoicacid	$C_{19} H_{36} O_2$	296.27083	1.47303	0.00522	2.8013998
Negative	M_753	(R)-Lactate	$C_3 H_6 O_3$	90.03137	1.38564	0.003886	1.5262159
Negative	M_759	Catechol	$C_6 H_6 O_2$	110.03533	1.4527	0.0061855	0.0845404
Negative	M_760	Kynurenic acid	$C_{10} H_7 N O_3$	189.04158	1.16345	0.0268248	0.4060352
Negative	M_788	felbamate	$C_{11} H_{14} N_2 O_4$	238.0946	1.40802	0.0082056	0.3921548
Negative	M_797	Benzoic acid	$C_7 H_6 O_2$	122.03539	1.3801	0.0228189	2.4427619
Negative	M_803	octyl methoxycinnamate	$C_{18} H_{26} O_3$	290.18772	1.42998	0.0057107	0.5010576
Negative	M_836	3-Deoxy-D-manno-octulosonate	$C_8 H_{14} O_8$	238.0682	1.32276	0.0368925	1.5901839
Negative	M_844	L-Dopa	$C_9 H_{11} N O_4$	197.06782	1.2499	0.0182086	0.5656176
Negative	M_857	MAB-CHMINACA metabolite M3	$C_{21} H_{29} N_3 O_4$	387.21131	1.14988	0.0263497	0.6817131
Negative	M_923	(S)-2-Acetolactate	$C_5 H_8 O_4$	132.04198	1.67011	6.67E-05	2.6793467
Negative	M_959	2-Hydroxycinnamic acid	$C_9 H_8 O_3$	164.04613	1.24989	0.0375998	0.6801452
Negative	M_972	2-amino-2,3,7-trideoxy-D-lyxo-hept-6-ulosonic acid	$C_7 H_{13} N O_5$	191.07843	1.33116	0.0391011	1.5499024
Negative	M_985	Leu-arg	$C_{12} H_{25} N_5 O_3$	287.19545	1.5577	0.002943	0.4517054
Negative	M_1031	MFCD03411993	$C_{18} H_{30} O_5$	326.20896	1.32479	0.0179095	1.732001
Negative	M_1039	3-Ketolactose	$C_{12} H_{20} O_{11}$	340.09987	1.52984	0.0014435	2.3782457
Negative	M_1053	Succinyl proline	$C_9 H_{13} N O_5$	215.07836	1.21396	0.0240299	0.5807237
Negative	M_1127	(-)-Shikimic acid	$C_7 H_{10} O_5$	174.05179	1.34667	0.0338552	1.6742872
Negative	M_1160	trans-Aconitic acid	$C_6 H_6 O_6$	174.0153	1.3166	0.0344069	1.4274191
Negative	M_1162	Gly-Arg	$C_8 H_{17} N_5 O_3$	231.13232	1.25447	0.0469	0.3762723

Negative	M_1178	Valylproline	$C_{10}H_{18}N_2O_3$	214.13086	1.21447	0.0337983	0.6695641
Negative	M_1189	4-[(2-Isopropyl-5-methylcyclohexyl)oxy]-4-oxobutanoic acid	$C_{14}H_{24}O_4$	256.16692	1.36565	0.0247238	2.1894502
Negative	M_1205	2,3,4,5-tetrahydrodipicolinic acid	$C_7H_9NO_4$	171.05224	1.27149	0.0320898	1.4855972
Negative	M_1232	Eglumetad	$C_8H_{11}NO_4$	185.06795	1.23142	0.0431037	1.6132845
Negative	M_1251	5D-5-O-Methyl-2_3_5/4_6-pentahydroxycyclohexanone	$C_7H_{12}O_6$	192.06396	1.12157	0.046183	0.6392911
Negative	M_1260	N_N-Dihydroxy-L-tyrosine	$C_9H_{11}NO_5$	213.06277	1.21084	0.0335588	0.4936535
Negative	M_1333	L-Alanyl-L-proline	$C_8H_{14}N_2O_3$	186.09954	1.15719	0.02079	0.6845465
Negative	M_1336	gamma-L-Glutamylputrescine	$C_9H_{19}N_3O_3$	217.14179	1.25955	0.0255521	0.6821428
Negative	M_1393	3-oxopalmitic acid	$C_{16}H_{30}O_3$	270.21907	1.24922	0.0240248	1.9954056
Negative	M_1424	7-Acetoxy-4-hydroxy-N,N,N-trimethyl-10-oxo-3,5,9-trioxa-4-phosphapentacosan-1-aminium 4-oxide	$C_{26}H_{53}NO_8P$	538.3484	1.5024	0.0081619	0.4533188
Negative	M_1567	4-Acetamido-3-hydroxyphenyl hydrogen sulfate	$C_8H_9NO_6S$	247.01465	1.27656	0.027646	0.1258957
Negative	M_1582	N-glycoloyl-D-mannosaminolactone	$C_8H_{13}NO_7$	235.06851	1.37893	0.0158648	1.6252411
Negative	M_1595	Neuraminicacid	$C_9H_{17}NO_8$	267.09466	1.6556	1.36E-06	1.7849341
Negative	M_1605	Guanine	$C_5H_5N_5O$	151.04816	1.42705	0.0040198	0.3249113
Negative	M_1646	(3β,5ξ,9ξ,18ξ)-28-Hydroxy-28-oxoolean-12-en-3-yl 6-deoxy-α-L-mannopyranosyl-(1->3)-[β-D-glucopyranosyl-(1->2)]-β-D-glucopyranosiduronic acid	$C_{48}H_{76}O_{18}$	940.50123	1.41845	0.0129297	1.8999141
Negative	M_1718	β-Estradiol-17β-glucuronide	$C_{24}H_{32}O_8$	448.21257	1.07103	0.0478764	0.6326312
Negative	M_1752	RH-34	$C_{18}H_{19}N_3O_3$	650.27851	1.22276	0.0227476	0.5385062
Negative	M_1760	11343172	$C_{11}H_{21}NO_3$	215.15131	1.34162	0.0222484	1.8035783
Negative	M_1793	(Ac)2-L-Lys-D-Ala	$C_{13}H_{23}N_3O_5$	301.16344	1.01178	0.031027	0.7645057

Negative	M_1835	fominoben	$C_{21} H_{24} Cl N_3 O_3$	401.15245	1.34571	0.0098692	1.6125197
Negative	M_1838	lys-met	$C_{11} H_{23} N_3 O_3 S$	277.14551	1.1641	0.0318397	0.5295516
Negative	M_1841	5?-androstane-3?,17?-diol dipropionate	$C_{25} H_{40} O_4$	404.292	1.24088	0.0138862	0.5283003
Negative	M_1855	arg-ala	$C_9 H_{19} N_5 O_3$	245.14807	1.52627	0.0014316	0.3966571
Negative	M_1924	(E)-4-hydroxyphenylacetaldehydeoxime	$C_8 H_9 N O_2$	151.06317	1.58436	0.0008857	0.3291275
Negative	M_1964	arg-val	$C_{11} H_{23} N_5 O_3$	273.17968	1.52701	0.0017465	0.3202776
Negative	M_1983	Butabarbital	$C_{10} H_{16} N_2 O_3$	212.11539	1.18927	0.044003	1.6799928
Negative	M_2023	Fortimicin FU-10	$C_{12} H_{24} N_2 O_9$	340.14878	1.36483	0.0079182	0.1451937
Negative	M_2035	Imidazoleacetic acid	$C_5 H_6 N_2 O_2$	126.04158	1.16217	0.0441165	0.5399883
Negative	M_2043	Trepibutone	$C_{16} H_{22} O_6$	310.14121	1.40696	0.0317301	1.9491287
Negative	M_2051	3a,7a,12a,24-Tetrahydroxy-5b-cholestan-26-oic acid	$C_{27} H_{46} O_6$	466.32882	1.16552	0.037594	0.5756782
Negative	M_2128	Pentobarbital	$C_{11} H_{18} N_2 O_3$	226.13111	1.23249	0.048836	1.5686454
Negative	M_2152	2762577	$C_{15} H_{23} N_3 O_4$	309.16847	1.19265	0.0466846	0.5452694
Negative	M_2164	Dexamethasone beloxil	$C_{29} H_{35} F O_5$	482.24846	1.15355	0.0380594	0.4790035
Negative	M_2242	trp-lys	$C_{17} H_{24} N_4 O_3$	332.18468	1.26575	0.0285794	0.4232537
Negative	M_2404	4-Hydroxy-5-methoxy-4-[2-methyl-3-(3-methyl-2-buten-1-yl)-2-oxiranyl]-1-oxaspiro[2.5]octan-6-one	$C_{16} H_{24} O_5$	296.16196	1.21528	0.0367443	0.4307484
Negative	M_2423	6-Hydroxy-5-methyl-4,11-dioxoundecanoic acid	$C_{12} H_{20} O_5$	244.13051	1.2073	0.026854	1.5107704
Negative	M_2488	alpha-L-Glucopyranosyl-(1->4)-alpha-L-glucopyranosyl-(1->4)-alpha-L-glucopyranosyl-(1->4)-alpha-L-glucopyranosyl-(1->4)-alpha-L-glucopyranosyl-(1->4)-alpha-D-glucopyranosyl-(1->4)-alpha-D-glucopyranos	$C_{54} H_{92} O_{46}$	1476.48221	1.31697	0.0268674	2.3040685

		yl-(1->4)-alpha-D-glucopyranosyl-(1->4)-D-glucopyranose					
Negative	M_2576	Methyl alpha-aspartylphenylalaninate	$C_{14} H_{18} N_2 O_5$	294.12133	1.44797	0.0179161	2.1099861
Negative	M_2594	Oxagrelate	$C_{14} H_{16} N_2 O_4$	276.1104	1.40201	0.024059	2.31669
Negative	M_2597	3-[(2Z)-1-Oxo-2-buten-2-yl]pentanedioic acid	$C_9 H_{12} O_5$	200.06753	1.19471	0.0344651	1.6975049
Negative	M_2622	2_6-Dihydroxypseudooxynicotine	$C_{10} H_{14} N_2 O_3$	210.09966	1.08916	0.0340978	0.6343668
Positive	M_3956	Methylglyoxal	$C_3 H_4 O_2$	72.02135	1.64225	0.001402	2.0593191
Positive	M_94	allopurinol	$C_5 H_4 N_4 O$	136.03796	1.62036	0.0002031	0.1509734
Positive	M_624	Xanthine	$C_5 H_4 N_4 O_2$	152.03275	1.61586	7.91E-05	0.1489807
Positive	M_2999	1-(beta-D-Ribofuranosyl)-1,2-dihydropyrimidine	$C_9 H_{14} N_2 O_4$	214.09438	1.60161	0.0011318	1.9417811
Positive	M_3852	1,3,5-Heptatriene	$C_7 H_{10}$	94.07804	1.57016	0.0013571	1.6009474
Positive	M_1337	Thymidine	$C_{10} H_{14} N_2 O_5$	242.08913	1.55159	0.0100447	1.4308911
Positive	M_2115	2-Hydroxy-2_4-pentadienoate	$C_5 H_6 O_3$	114.03129	1.54805	0.002528	2.0808006
Positive	M_3315	Prazepam	$C_{19} H_{17} Cl N_2 O$	324.10397	1.54598	0.0029626	1.7753001
Positive	M_3044	3-Hydroxy-2-methyl-4-pyrone	$C_6 H_6 O_3$	126.03147	1.54469	0.0058276	1.6629204
Positive	M_1182	Lys-Pro	$C_{11} H_{21} N_3 O_3$	243.15712	1.53989	0.0001596	0.5570408
Positive	M_765	4-Hydroxy-2-butynal	$C_4 H_4 O_2$	84.02119	1.53914	0.0028132	2.2642015
Positive	M_2933	Anisole	$C_7 H_8 O$	108.0573	1.53736	0.0008639	0.5226162
Positive	M_938	(S)-Methylmalonatesemialdehyde	$C_4 H_6 O_3$	102.03179	1.53612	0.002682	2.6296823
Positive	M_2061	Dinoseb	$C_{10} H_{12} N_2 O_5$	240.07343	1.53342	0.0033653	1.5518932
Positive	M_3341	9_12-Dioxododecanoicacid	$C_{12} H_{20} O_4$	228.13505	1.51798	0.0105431	2.1713569
Positive	M_1776	trans-2-Tetradecenoylcarnitine	$C_{21} H_{39} N O_4$	369.28624	1.51736	0.001887	2.3905797
Positive	M_1493	EJ4025000	$C_8 H_{18} N_2 O$	158.14128	1.5115	0.0023618	0.2009383
Positive	M_185	(2R_3S)-2_3-Dimethylmalate	$C_6 H_{10} O_5$	162.05208	1.50724	0.0044922	2.657246

Positive	M_3219	(R)-Pantolactone	$C_6 H_{10} O_3$	130.06241	1.5062	0.0108057	1.7349037
Positive	M_1804	N(6)-[(Indol-3-yl)acetyl]-L-lysine	$C_{16} H_{21} N_3 O_3$	303.15701	1.50576	0.0027244	0.4439258
Positive	M_534	3_4-Dehydrothiomorpholine-3-carboxylate	$C_5 H_7 N O_2 S$	145.0192	1.50092	0.0047727	0.092455
Positive	M_3245	2-(1,2-Dihydroxy-2-propanyl)-9-hydroxy-6,10-dimethylspiro[4.5]dec-6-en-8-one	$C_{15} H_{24} O_4$	268.16613	1.48232	0.0159193	2.4220215
Positive	M_1536	dihydroxyphenylalanine	$C_9 H_{11} N O_4$	197.06773	1.48173	0.0039948	1.8341772
Positive	M_2872	YQ3683000	$C_{11} H_{18} O_2$	182.12978	1.48012	0.0127906	1.6862778
Positive	M_2535	3-Hydroxy-4-methylanthranilate	$C_8 H_9 N O_3$	167.05741	1.47666	0.0083419	2.0822252
Positive	M_4010	3909	$C_6 H_{10} O$	98.07266	1.47544	0.0325507	1.6163079
Positive	M_3779	N-Feruloyltyramine	$C_{18} H_{19} N O_4$	313.13292	1.4673	0.0087014	3.2198448
Positive	M_1221	[FA(18:4)]6Z_9Z_12Z_15Z-octadecatetraenoicacid	$C_{18} H_{28} O_2$	276.20757	1.4627	0.0194046	2.0419007
Positive	M_752	1-Carbapen-2-em-3-carboxylicacid	$C_7 H_7 N O_3$	153.04187	1.4618	0.0061974	1.7514993
Positive	M_2125	Sulcatone	$C_8 H_{14} O$	126.1041	1.45541	0.0173865	1.5580154
Positive	M_3382	C7-HSL	$C_{11} H_{19} N O_3$	213.13551	1.4553	0.0088507	2.4748035
Positive	M_1779	Decylubiquinol	$C_{19} H_{32} O_4$	324.2285	1.454	0.0079445	2.3995166
Positive	M_2460	3-Allylcyclohexene	$C_9 H_{14}$	122.1091	1.45274	0.0140536	1.6847924
Positive	M_2065	(4S)-4-[(6-Carboxyhexanoyl)oxy]-4-(trimethylammonio)butanoate	$C_{14} H_{25} N O_6$	303.16807	1.44654	0.0096069	1.8364629
Positive	M_719	3-Epihydroxy-2'-deoxymugineicacid	$C_{12} H_{20} N_2 O_8$	320.12052	1.44527	0.0497311	1.5441177
Positive	M_2083	Remifentanil	$C_{20} H_{28} N_2 O_5$	376.19946	1.44346	0.0188653	2.6826508
Positive	M_1887	Apigenin7-(6''-malonylglucoside)	$C_{24} H_{22} O_{13}$	518.10388	1.44305	0.0455155	1.594125
Positive	M_79	VS1150000	$C_{18} H_{34} O_4$	314.24407	1.44111	0.0035892	1.6059679
Positive	M_803	Clavulanicacid	$C_8 H_9 N O_5$	199.04708	1.44001	0.0128409	1.3039618
Positive	M_3860	2-Methyl-3-hydroxy-5-formylpyridine-4-carboxylate	$C_8 H_7 N O_4$	181.03686	1.43963	0.0156426	3.8367051
Positive	M_1528	Ibuprofen	$C_{13} H_{18} O_2$	206.12987	1.43423	0.0115432	1.663882

Positive	M_3032	3-Hydroxytetradecanedioic acid	$C_{14} H_{26} O_5$	274.17687	1.43267	0.0185621	1.894671
Positive	M_2488	mebutamate	$C_{10} H_{20} N_2 O_4$	232.14137	1.42871	0.0452807	1.6268123
Positive	M_1954	Lys-Arg	$C_{12} H_{26} N_6 O_3$	302.20747	1.42531	0.0135451	1.5245612
Positive	M_2547	3413	$C_{11} H_{16} O$	164.11925	1.414	0.0231941	1.6213132
Positive	M_1504	4-(Trimethylammonio)-3-(undecanoyloxy)butanoate	$C_{18} H_{35} N O_4$	329.25494	1.41079	0.03262	1.7626375
Positive	M_3364	(-)-Epothilone D	$C_{27} H_{41} N O_5 S$	491.27014	1.40925	0.0112322	1.9072598
Positive	M_2815	NPPB	$C_{16} H_{16} N_2 O_4$	300.1098	1.40703	0.0388215	2.187264
Positive	M_2451	9-Oxononanoicacid	$C_9 H_{16} O_3$	172.1092	1.40342	0.0372673	1.5839279
Positive	M_1888	His-Trp	$C_{17} H_{19} N_5 O_3$	341.14752	1.40183	0.0098694	0.5857085
Positive	M_2424	2-Hydroxyfelbamate	$C_{11} H_{14} N_2 O_5$	254.08875	1.40169	0.0162085	1.6702191
Positive	M_859	Furfural;Furan-2-carbaldehyde	$C_5 H_4 O_2$	96.02087	1.40161	0.0183243	1.7453815
Positive	M_2260	3213	$C_9 H_{16} O$	140.11966	1.40075	0.0308964	1.5722747
Positive	M_500	Kojic acid	$C_6 H_6 O_4$	142.02584	1.3982	0.0186054	2.0401319
Positive	M_3248	Prenyl-L-cysteine	$C_8 H_{15} N O_2 S$	189.08156	1.39587	0.0066502	0.2948957
Positive	M_3294	Catechol;1_2-Dihydroxybenzene	$C_6 H_6 O_2$	110.03652	1.39534	0.0142724	2.3122398
Positive	M_251	I-Urobilinogen	$C_{33} H_{44} N_4 O_6$	592.32334	1.39294	0.0014806	0.4002687
Positive	M_3556	procainamide	$C_{13} H_{21} N_3 O$	235.16745	1.39022	0.0228589	3.1256431
Positive	M_557	5-O-methyl embelin	$C_{18} H_{28} O_4$	308.19731	1.38813	0.0181488	1.9609043
Positive	M_3702	L-Carnitinamide	$C_7 H_{16} N_2 O_2$	160.12044	1.38588	0.0004849	0.7403009
Positive	M_109	[FA(18:0)]12R_13S-epoxy-9Z-octadecenoicacid	$C_{18} H_{32} O_3$	296.2337	1.38581	0.0088953	1.4732078
Positive	M_3805	Metalaxyl	$C_{15} H_{21} N O_4$	279.14598	1.38565	0.0382371	1.6411473
Positive	M_3753	[FA(6:1)]2-hexenal	$C_6 H_{10} O$	98.07323	1.38392	0.0294882	1.5759525
Positive	M_191	6-Acetamido-3-aminohexanoate	$C_8 H_{16} N_2 O_3$	188.11532	1.38367	0.0071591	0.6594158
Positive	M_3076	5-Valerolactone	$C_5 H_8 O_2$	100.05225	1.38258	0.0424443	1.6096972

Positive	M_455	4-Guanidinobutanal	$C_5 H_{11} N_3 O$	129.08976	1.37894	0.0250392	1.9125631
Positive	M_1817	N5-Ethyl-L-glutamine	$C_7 H_{14} N_2 O_3$	174.09979	1.37584	0.0106721	0.4915315
Positive	M_1505	D-Arabitol	$C_5 H_{12} O_5$	152.06779	1.37332	0.0219101	1.710328
Positive	M_585	[FAhydroxy(4:0)]N-(3S-hydroxy-butanoyl)-homoserinelactone	$C_8 H_{13} N O_4$	187.08357	1.37309	0.0318435	1.8228721
Positive	M_3604	(2E_6E)-(10R_11S)-10_11-Epoxy-3_7_11-trimethyltrideca-2_6-dienoicacid	$C_{16} H_{26} O_3$	266.18691	1.37185	0.0336871	2.1165091
Positive	M_72	2-(6'-methylthio)hexylmalate	$C_{11} H_{20} O_5 S$	264.1031	1.36868	0.0267205	2.6445532
Positive	M_3288	(2S)-1-[(9Z)-9-Hexadecenoyloxy]-3-hydroxy-2-propanyl (9Z)-9-heptadecenoate	$C_{36} H_{66} O_5$	578.48826	1.36851	0.0191059	2.9039643
Positive	M_331	Thiomorpholine3-carboxylate	$C_5 H_9 N O_2 S$	147.03472	1.3678	0.0092052	0.1119584
Positive	M_1500	1_2-Benzoquinone	$C_6 H_4 O_2$	108.02081	1.36673	0.0213811	2.294619
Positive	M_1748	cyclandelate	$C_{17} H_{24} O_3$	276.17142	1.36469	0.0024078	0.5906438
Positive	M_409	5-(2-Hydroxyethyl)-4-methylthiazole	$C_6 H_9 N O S$	143.0399	1.35809	0.0297766	2.6149348
Positive	M_1053	Hydroxy-1,4-benzoquinone	$C_6 H_4 O_3$	124.01557	1.35594	0.0319328	1.7525675
Positive	M_491	2_3-Dimethylmaleate	$C_6 H_8 O_4$	144.04164	1.35466	0.0296928	2.902559
Positive	M_1305	2-Hydroxyphenethylamine	$C_8 H_{11} N O$	137.08376	1.35228	0.0055167	0.3001199
Positive	M_1089	9-KODE	$C_{18} H_{30} O_3$	294.21799	1.35053	0.04227	1.9495933
Positive	M_748	Deoxyuridine	$C_9 H_{12} N_2 O_5$	228.07368	1.34356	0.0079989	0.3386791
Positive	M_1460	Zalcitabine	$C_9 H_{13} N_3 O_3$	211.09476	1.34341	0.0082865	0.2802682
Positive	M_1209	2-Formylglutarate	$C_6 H_8 O_5$	160.03641	1.34192	0.0298912	1.5994073
Positive	M_212	4-Amino-5-hydroxymethyl-2-methylpyrimidine	$C_6 H_9 N_3 O$	139.0742	1.33698	0.0341928	2.5201469
Positive	M_96	Coumarone	$C_8 H_6 O$	118.0414	1.3358	0.0204453	0.5675914
Positive	M_3497	Marinobufagenin	$C_{24} H_{32} O_5$	400.22531	1.3358	0.0386298	2.3585427
Positive	M_95	2-Aminoacetophenone;O-Acetylaniline	$C_8 H_9 N O$	135.06782	1.33491	0.0206164	0.5679751

Positive	M_680	Indole;1-Benzazole	$C_8 H_7 N$	117.05752	1.33405	0.0039036	0.6711922
Positive	M_728	N-((4-AMINO-2-METHYL-5-PYRIMIDINYL)METHYL)FORMAMIDE	$C_7 H_{10} N_4 O$	166.08496	1.32563	0.0326115	4.3227825
Positive	M_3221	2_6-Diaminoheptanedioicacid	$C_7 H_{14} N_2 O_4$	190.0945	1.32188	0.0263214	0.275394
Positive	M_726	1_2-Diaminobenzene	$C_6 H_8 N_2$	108.06847	1.32186	0.0102105	0.2782715
Positive	M_1202	3-Carboxy-1-methylpyridinium	$C_7 H_7 N O_2$	137.04709	1.32153	0.0307183	2.0995132
Positive	M_1228	Uracil;2_4-Dihydroxypyrimidine	$C_4 H_4 N_2 O_2$	112.02711	1.31926	0.0094193	0.4333908
Positive	M_399	3-[18-(3-Methoxy-3-oxopropyl)-3,7,12,17-tetramethyl-8,13-divinyl-2-porphyrinyl]propanoic acid	$C_{35} H_{36} N_4 O_4$	576.27083	1.31675	0.0352769	3.1043954
Positive	M_139	(3S)-3_6-Diaminohexanoate	$C_6 H_{14} N_2 O_2$	146.10489	1.31257	0.0144087	0.5838218
Positive	M_2649	tranexamic acid	$C_8 H_{15} N O_2$	157.10973	1.31185	0.0409096	2.5047276
Positive	M_22	2-coumarate	$C_9 H_8 O_3$	164.04667	1.31153	0.0281222	0.5912364
Positive	M_2975	MFCD00025555	$C_9 H_{18} N_2 O_3$	202.13091	1.31118	0.0239938	0.4273103
Positive	M_3409	tilisolol	$C_{17} H_{24} N_2 O_3$	304.17749	1.30772	0.0019686	0.4355575
Positive	M_23	3-Amino-3-(4-hydroxyphenyl)propanoate	$C_9 H_{11} N O_3$	181.07314	1.30728	0.0292961	0.5861343
Positive	M_196	D-Pipecolicacid	$C_6 H_{11} N O_2$	129.07838	1.30367	0.0187724	0.5557238
Positive	M_1086	Styrene	$C_8 H_8$	104.06229	1.2993	0.005729	0.6759882
Positive	M_1602	8-methylthiooctanaldoxime	$C_9 H_{19} N O S$	189.11862	1.29561	0.0139288	0.6854193
Positive	M_939	(7E,13Z)-3-Benzyl-6,12-dihydroxy-4,5,10,12-tetramethyl-1-oxo-2,3,3a,6,6a,9,10,11,12,15-decahydro-1H-cycloundeca[d]isoindol-15-yl acetate	$C_{30} H_{39} N O_5$	493.28304	1.29205	0.0035956	0.7026337
Positive	M_2714	N,N-Bis(2-hydroxyethyl)dodecanamide	$C_{16} H_{33} N O_3$	287.24457	1.28228	0.0086183	0.4981676
Positive	M_2860	3-Hydroxy-3-[(3-methylbutanoyl)oxy]-4-(trimethylammonio)butanoate	$C_{12} H_{23} N O_5$	261.15654	1.28221	0.0114264	0.5517985
Positive	M_3563	apronalide	$C_9 H_{16} N_2 O_2$	184.12047	1.28175	0.049572	1.990688

Positive	M_1930	1-Methylpyrrolinium	$C_5 H_9 N$	83.07366	1.28062	0.0239368	0.5337663
Positive	M_721	2-methylhistamine	$C_6 H_{11} N_3$	125.09498	1.27958	0.0137884	0.3101602
Positive	M_1128	5-Methylcytosine	$C_5 H_7 N_3 O$	125.05851	1.27659	0.0084587	0.1887206
Positive	M_2946	lys-thr	$C_{10} H_{21} N_3 O_4$	247.15249	1.27452	0.0111448	0.595837
Positive	M_225	Lysylvaline	$C_{11} H_{23} N_3 O_3$	245.17281	1.27158	0.0331613	0.4060341
Positive	M_2132	L-Tyrosinemethylester	$C_{10} H_{13} N O_3$	195.08879	1.27084	0.0191275	0.3874594
Positive	M_1865	4-Deoxy-5-C-(3,5-di-sec-butyl-1-cyclopenten-1-yl)pentonic acid	$C_{18} H_{32} O_5$	328.22349	1.26499	0.0470972	2.5506683
Positive	M_903	Naphthalene	$C_{10} H_8$	128.06199	1.26313	0.0105183	0.6507008
Positive	M_100	949521	$C_{29} H_{35} N O_5$	477.25232	1.25832	0.0237313	1.7975212
Positive	M_430	Pyrrolidine	$C_4 H_9 N$	71.07379	1.24325	0.0324388	0.3933549
Positive	M_1173	2'-Deoxyinosine	$C_{10} H_{12} N_4 O_4$	252.0847	1.24254	0.0202153	0.4918878
Positive	M_551	Linoleamide	$C_{18} H_{33} N O$	279.25487	1.24086	0.0130078	0.5794559
Positive	M_235	4-Hydroxybenzaldehyde	$C_7 H_6 O_2$	122.03641	1.22389	0.023969	0.6192339
Positive	M_1910	Gly-Lys	$C_8 H_{17} N_3 O_3$	203.12603	1.21953	0.0298074	0.5395225
Positive	M_1929	ala-met	$C_8 H_{16} N_2 O_3 S$	220.08715	1.21363	0.0416207	0.3132
Positive	M_86	3-Oxo-5beta-cholanate	$C_{24} H_{38} O_3$	374.2804	1.20342	0.0413643	0.147662
Positive	M_1909	Nicotinuricacid	$C_8 H_8 N_2 O_3$	180.05277	1.19865	0.0290853	0.0727846
Positive	M_3430	Pronilin	$C_{21} H_{29} Cl O_6 S$	444.13523	1.19703	0.038107	0.4648385
Positive	M_2676	6-Hydroxypseudooxynicotine	$C_{10} H_{14} N_2 O_2$	194.10483	1.18638	0.0092078	0.6629542
Positive	M_3901	3,4a,5-Trimethyl-6-{[(2E)-3-(methylsulfanyl)-2-propenoyl]oxy}-4,4a,5,6,7,8,8a,9-octahydronaphtho[2,3-b]furan-4-yl (2E)-2-methyl-2-butenoate	$C_{24} H_{32} O_5 S$	432.19896	1.18226	0.0137068	0.7300793

Positive	M_2081	4a-Carbinolaminetetrahydrobiopterin	$C_9 H_{13} N_5 O_3$	239.1007	1.18155	0.0246338	0.4807282
Positive	M_1162	Alfentanil	$C_{21} H_{32} N_6 O_3$	416.25548	1.17846	0.0419763	0.5299542
Positive	M_848	Val-Met	$C_{10} H_{20} N_2 O_3 S$	248.11844	1.1778	0.0479072	0.3865102
Positive	M_304	lysophosphatidylethanolamine (0:0/22:6(4Z,7Z,10Z,13Z,16Z,19Z))	$C_{27} H_{44} N O_7 P$	525.28365	1.17615	0.0387273	0.4774418
Positive	M_3923	gamma-Glutamylleucine	$C_{11} H_{20} N_2 O_5$	260.13628	1.17361	0.0131826	0.7073109
Positive	M_2425	Benzaldehyde	$C_7 H_6 O$	106.0417	1.1714	0.0360975	0.4817105
Positive	M_393	Coumarin	$C_9 H_6 O_2$	146.03614	1.17054	0.0405864	0.6048656
Positive	M_1802	D-Lysyl-L-valyl-D-valyl-L-allothreonine	$C_{20} H_{39} N_5 O_6$	445.288	1.16112	0.0399858	0.4624204
Positive	M_1012	Voglibose	$C_{10} H_{21} N O_7$	267.13141	1.16081	0.0296556	0.6849784
Positive	M_1832	3-Methylcyclohexanethiol	$C_7 H_{14} S$	130.08169	1.15774	0.0492216	0.5429965
Positive	M_2810	Cystathionine ketimine	$C_7 H_9 N O_4 S$	203.02525	1.14103	0.0351067	0.4684166
Positive	M_1138	5-Methyldeoxycytidine	$C_{10} H_{15} N_3 O_4$	241.10517	1.13087	0.0459616	0.5539112
Positive	M_989	trimethadione	$C_6 H_9 N O_3$	143.05767	1.12644	0.0357519	0.6192756
Positive	M_566	LysoPC(22:5(7Z,10Z,13Z,16Z,19Z))	$C_{30} H_{52} N O_7 P$	569.34501	1.12249	0.0362801	0.4893665
Positive	M_1761	Phenol	$C_6 H_6 O$	94.04184	1.11128	0.022138	0.8294394
Positive	M_2436	Levetiracetam	$C_8 H_{14} N_2 O_2$	170.10475	1.10753	0.0386682	0.7328995
Positive	M_2944	Ubiquinone-2(CoQ2)	$C_{19} H_{26} O_4$	318.18163	1.09967	0.029233	0.6285739
Positive	M_1006	N-Acetyl-L-methionine	$C_7 H_{13} N O_3 S$	191.06083	1.09755	0.0198425	0.7571691
Positive	M_633	7-Aminomethyl-7-deazaguanine	$C_7 H_9 N_5 O$	179.0799	1.09655	0.0488492	0.5462468
Positive	M_2598	513992	$C_{14} H_{22} O$	206.16606	1.09138	0.0319043	0.4975474
Positive	M_3471	2949	$C_{12} H_{22} N_2 O_4$	258.15679	1.09133	0.0306395	0.3474101
Positive	M_2548	ionene	$C_{13} H_{18}$	174.14011	1.08443	0.0247372	0.4919252
Positive	M_2572	Hexanoylglycine	$C_8 H_{15} N O_3$	173.10457	1.07337	0.0043566	0.7459654

Positive	M_2576	nivazol	$C_{28} H_{31} F N_2 O$	430.24071	1.07185	0.0462968	0.6492392
Positive	M_1172	Tetroxoprim	$C_{16} H_{22} N_4 O_4$	334.16261	1.05697	0.0480606	0.7284832
Positive	M_1845	Nipradilol	$C_{15} H_{22} N_2 O_6$	326.14635	1.02385	0.0392183	0.8323937

Note: FC: Fold change. The value of FC greater than 1 indicates up-regulated metabolites, and that of less than 1 indicates down-regulated metabolites. MW: Molecular weight; VIP: Variable importance in the projection value. OVX group: Ovariectomized rats are subjected to bilateral ovariectomy and treated with physiological saline; OVX-CS group: Ovariectomized rats are treated with CS (150 mg per kg body weight).
